# High-Performance Liquid Chromatography with Diode Array Detector and Electrospray Ionization Ion Trap Time-of-Flight Tandem Mass Spectrometry to Evaluate Ginseng Roots and Rhizomes from Different Regions

**DOI:** 10.3390/molecules21050603

**Published:** 2016-05-09

**Authors:** Hong-Ping Wang, You-Bo Zhang, Xiu-Wei Yang, Xin-Bao Yang, Wei Xu, Feng Xu, Shao-Qing Cai, Ying-Ping Wang, Yong-Hua Xu, Lian-Xue Zhang

**Affiliations:** 1 State Key Laboratory of Natural and Biomimetic Drugs, Department of Natural Medicines, School of Pharmaceutical Sciences, Peking University Health Science Center, Peking University, No. 38, Xueyuan Road, Haidian District, Beijing 100191, China; sungirl9626@163.com (H.-P.W.); zybo5288@163.com (Y.-B.Z.); xbyang0718@163.com (X.-B.Y.); high-xu@163.com (W.X.); xufeng_pharm@163.com (F.X.); sqcai@bjmu.edu.cn (S.-Q.C.); 2 Institute of Special Wild Economic Animals and Plants Science, Chinese Academy of Agricultural Sciences, Changchun 130112, China; yingpingw@yeah.net; 3 College of Chinese Medicinal Materials, Jilin Agricultural University, Changchun 130118, China; xuyonghua777@yeah.net (Y.-H.X.); zlx863@163.com (L.-X.Z.)

**Keywords:** *Panax ginseng*, ginsenosides, mass spectrometry, chemical substances

## Abstract

Ginseng, *Panax ginseng* C. A. Meyer, is an industrial crop in China and Korea. The functional components in ginseng roots and rhizomes are characteristic ginsenosides. This work developed a new high-performance liquid chromatography coupled with electrospray ionization ion trap time-of-flight multistage mass spectrometry (LC–ESI-IT-TOF-MS^n^) method to identify the triterpenoids. Sixty compounds (**1**–**60**) including 58 triterpenoids were identified from the ginseng cultivated in China. Substances **1**, **2**, **7**, **15**–**20**, **35**, **39**, **45**–**47**, **49**, **55**–**57**, **59**, and **60** were identified for the first time. To evaluate the quality of ginseng cultivated in Northeast China, this paper developed a practical liquid chromatography–diode array detection (LC–DAD) method to simultaneously quantify 14 interesting ginsenosides in ginseng collected from 66 different producing areas for the first time. The results showed the quality of ginseng roots and rhizomes from different sources was different due to growing environment, cultivation technology, and so on. The developed LC–ESI-IT-TOF-MS^n^ method can be used to identify many more ginsenosides and the LC–DAD method can be used not only to assess the quality of ginseng, but also to optimize the cultivation conditions for the production of ginsenosides.

## 1. Introduction

Ginseng, *Panax ginseng* C. A. Meyer of the *Araliaceae* family, is mainly distributed in Northeast China, Korea, and the border areas of Russia. Ginseng roots and rhizomes (GRR) are used as herbal drugs in traditional Chinese medicine for the treatment of neurological disorders and other diseases. In recent years, GRR has been increasingly used as a health tonic and food, in the form of a variety of commercial health products including ginseng capsules, teas, milk, chocolates, cookies, candy, and cosmetics, *etc.*. Public use of ginseng in the food field continues to grow. Nowadays, wild ginseng is rarely available and the GRR on the market are mostly collected from farms cultivating ginseng in fields. Ginseng is sold as a food additive in the U.S. and, thus, it need not meet specific safety and efficacy requirements of the Food and Drug Administration. It is estimated that the current world sales of various ginseng raw materials have reached over 300 billion US dollars per annum. Ginseng has been developed as a valuable industrial crop and is now widely used around the world. Good Agricultural Practice (GAP) bases of ginseng have been established in Jingyu, Changbai, Ji’an, Fusong counties and so on in Jilin province of China in recent years. Due to the influence of the factors such as environment and cultivation techniques, quality of GRR varies from different sources. Consensus opinion [1] suggests that the main bioactive principles of GRR are ginsenosides (ginseng saponins), derivatives of the triterpene dammarane and/or oleanolic acid structures, especially dammarane ones, which exhibit properties of anti-cancer [2,3,4,5], neuroprotective [5,6,7,8,9], anti-oxidant [5,10,11,12,13], hepatoprotective [5,14], anti-nociception [5,15], anti-inflammatory, anti-stress, hypoglycemic, anti-fatigue [5], melanogenesis inhibitory [16], and silent information regulator two homolog 1 activator [17] effects. In addition, some ginsenosides and their aglycone exhibit good pharmacokinetic properties [18,19]. To date, more than 50 ginseng saponins [1] have been isolated and unambiguously characterized from GRR. The dammarane-type saponins can be further divided into 20(*R*/*S*)-protopanaxadiol (PPD) and 20(*R*/*S*)-protopanaxatriol (PPT) groups according to their aglycones. Since the ginsenosides have been claimed to be responsible for the wide pharmacological responses of GRR, it is necessary to clarify its basic chemical substances. The analysis of ginsenosides faces great challenge because of the complexity and similarity of their chemical structures. With high resolution, sensitivity, and accuracy of mass analysis, the liquid chromatography-mass spectrometry (LC–MS) technology shows unique advantages in providing a large number of structural information of compounds. Today, LC–MS has been increasingly applied for analysis of the plant material to characterize the known compounds as well as to deduce other unknown compounds. For example, Zhang *et al.* [20] identified 25 ginsenosides in the red ginseng by liquid chromatography-electrospray ionization mass spectrometry (LC–ESI-MS/MS), and 28 ginsenosides were simultaneously characterized by LC–ESI-MS/MS method in another literature [21]. However, up to date, only a few of major ginsenosides in GRR have been identified, which are quite less than the number of ginsenosides indeed present. Compounds in minor or trace amounts have not been identified.

Nowadays, the cultivation districts of ginseng have been more than 60 regions in China. However, there are few reports to compare the GRR quality in these producing areas. In order to fully reflect the content of total saponins in GRR, enough saponins and regions of GRR should be supplied. As the main ingredients in GRR, the content of ginsenosides is an important index in assessing the quality of GRR. Many analytical approaches have been developed to quantify ginsenosides in the extracts of ginseng and its products, including LC coupled with an ultraviolet (UV) detector [22,23,24], or an evaporative light scattering detector (ELSD) [25,26,27]. Due to the complexity of the chemical constituents and the similarity of the numerous ginsenosides, many compounds may be co-eluted leading to inaccurate results. Diode array detector (DAD), which can be used to detect the peak-purity according to its spectrum, can be applied to accurately quantify ginsenosides in GRR.

This paper aims to develop a new and reliable liquid chromatography coupled with electrospray ionization ion trap time-of-flight multistage mass spectrometry (LC–ESI-IT-TOF-MS^n^) method to characterize the main and minor saponins in GRR for the first time. In addition, a practical LC–DAD method was developed to simultaneously determine the 14 major ginsenosides in GRR collected from 66 different regions. The quality of ginsenosides in GRR was comprehensively estimated in this paper and the differences of ginseng grown in China were clarified.

## 2. Results and Discussion

### 2.1. Structural Identification of Ginsenosides

**Figure 1:**
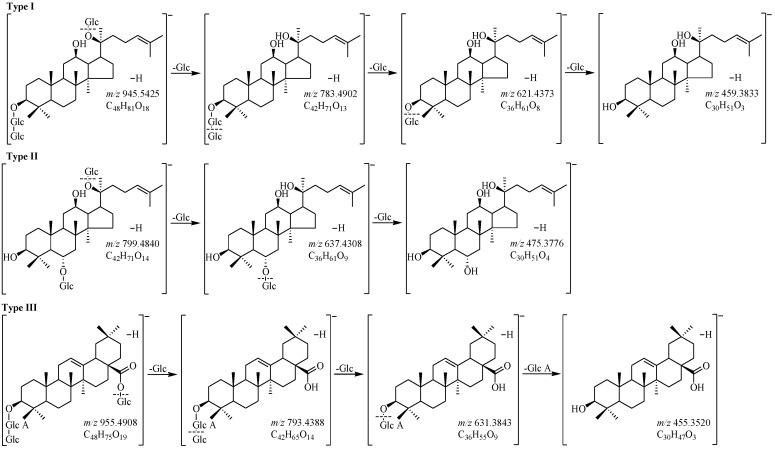
The fragmentation pathways of different types of ginsenosides: Type I [(20*S*)-protopanaxadiol], Type II [(20*S*)-protopanaxatriol], and Type III (oleanolic acid).

**Figure 2:**
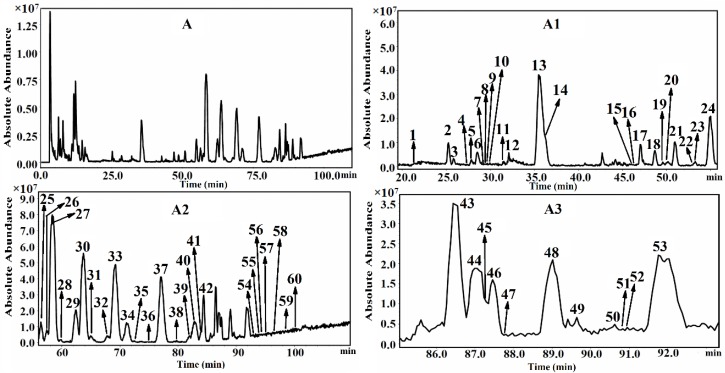
The TIC of Jilin GRR in negative ion detection mode (**A**) and its corresponding amplified chromatograms (**A1**, **A2**, **A3**).

Due to ginsenosides had not only higher sensitivity but also clearer mass spectra in the negative ion detection mode, the data of both the reference standards (Figure 1) and the samples (Figure 2) were acquired in negative-ion detection mode, which made it easier to detect ginsenosides of lower content and confirm molecular ions or quasi-molecular ions in the identification of each peak. According to the retention time (*t*_R_), ESI-MS (molecular weight), and MS/MS (fragment ions) information, the chromatographic behaviors and MS spectra of 32 reference standards were obtained (Table S1), which were the basis for identifying the other ingredients in GRR. The negative MS/MS spectra were obtained from the [M − H]^−^ ions, and they exhibited a fragmentation pattern corresponding to the successive loss of the glycosidic units until the formation of [aglycon − H]^−^ ions. According to the structural properties, PPD-type ginsenosides (type I), including **21**, **25**, **26**, **27**, **29**, **30**, **33**, **34**, **37**, **40**, **41**, **43**, **44**, and **58**, yielded an aglycone ion at *m*/*z* 459, while PPT-type ginsenosides (type II) possessed an aglycone ion at *m*/*z* 475 which was visible for **3**, **5**, **6**, **8**, **9**, **10**, **12**, **13**, **14**, **22**, **24**, **28**, **31**, and **32**. And oleanolic acid type ginsenosides (type III), including **50**, **53**, and **56**, produced an aglycone ion at *m*/*z* 455 (C_30_H_47_O_3_), corresponding to [oleanolic acid − H]^−^ (Figure 1). Therefore, the aglycones could be easily identified by finding these diagnostic fragment ions initially. The obtained neutral loss could be used to elucidate sugar moiety. The amount and the type of saccharide units were determined in which a mass difference of 162 indicated the presence of a glucosyl (Glc) group, while 132 indicated the presence of a pentosyl group [α-l-arabinose (Ara) (pyranose or furanose) or β-d-xylose (Xyl)]. A mass difference of 146 suggested the presence of an α-l-rhamnosyl (Rha) group, while 176 suggested the presence of a β-d-glucuronyl (Glu A) group. The obtained fragmentation pathways were used to identify the known ginsenosides that have been isolated from GRR or previously reported in the literatures and unknown ginsenosides that have not been reported to date.

Finally, a total of 58 triterpenoids were identified from GRR of Jilin province of China. The mass accuracy for all molecular ions showed a maximal deviation of 5 ppm from the theoretical mass, which made the characterization more reliable. The 32 ginsenosides described in Section 3.2 present in GRR were unambiguously characterized with LC–ESI-IT-TOF-MS^n^ by comparing with the *t*_R_ and fragmentation patterns of the reference standards. The others were tentatively assigned by matching the empirical molecular formulas and diagnostic fragment ions with those of the published known ginsenosides.

Peaks **35**, **39**, **42**, **45**, **46**, **48**, **49**, **51**, **52**, **54**, **55**, and **57** exhibited the fragment ions at *m*/*z* 459 corresponding to the PPD aglycone moiety, suggesting that they were the PPD-type ginsenosides. Ginsenoside (G)-Rb_1_ (peak **27**) was eluted at 58.45 min and the deprotonated molecular ion [M − H]^−^ was at *m*/*z* 1107. The fragment ions were observed at *m*/*z* 945 [M − H − Glc]^−^, 783 [M − H − 2Glc]^−^, 621 [M − H − 3Glc]^−^, and 459 [M − H − 4Glc]^−^. Peak **35** and quinquenoside (PQ)-R1 (peak **37**), peak **39** and malonyl (Ma)-G-Rb_1_ (peak **42**), peak **46** and G-Rd (peak **40**), peak **49** and Ma-G-Rc (peak **48**), peak **54** and pseudo-ginsenoside RC1 (pseudo-G-RC1) (peak **52**), as well as peak **57** and Ma-G-Rd (peak **55**) should be in each pair of isomers. Peak **45** (*t*_R_ 87.35 min) exhibited deprotonated molecular ion [M − H]^−^ at *m*/*z* 1163 and fragment ion at *m*/*z* 1119 [M − H − CO_2_]^−^. By matching the accurate masses and the fragment ions (Table S1) with those of a previous study [21], the peak **45** was assigned to be Ma-G-Rb_3_. Peak **51** (*t*_R_ 90.71 min) showed adduct ion [M + HCOO]^−^ at *m*/*z* 961.5356 and presented a low abundance deprotonated molecular ion [M − H]^−^ at *m*/*z* 915.5216, which indicated the molecular formula was C_47_H_80_O_17_. In the MS^2^ and MS^3^ spectra, the fragment ions [M − H − Xyl]^−^ at *m*/*z* 783.4865 and [M − H − Xyl − Glc]^−^ at *m*/*z* 621.4305 could be attributed to the successive loss of Xyl and Glc groups. Finally, the peak **51** was assigned to be vinaginsenoside (VG) R_16_, a ginsenoside isolated from the roots of ginseng [28].

Peaks **2**, **7**, **15**, **16**, **17**, **18**, **19**, **20**, **36**, **38**, and **47** exhibited the fragment ions at *m*/*z* 475 corresponding to the PPT aglycone moiety, suggesting that they were the PPT-type ginsenosides. Peak **2** and reference standard 20-glc-G-Rf (peak **6**) have the same deprotonated molecular ion [M − H]^−^ at *m*/*z* 961 and an adduct ion [M + HCOO]^−^ at *m*/*z* 1007 as well as fragment ions at *m*/*z* 799 [M − H − Glc]^−^, 637 [M − H − 2Glc]^−^, 475 [M − H − 3Glc]^−^, suggesting that they were a pair of isomers. Similarly, peak **7** and reference standard G-Re_4_ (peak **5**), peaks **15** and **16**, peaks **17** and **20**, peaks **36** and **38**, should be in each pair of isomers. By matching the accurate masses and the fragment ions (Table S1) with those of a previous study [21], peaks **19** and **47** were assigned to be acetyl-G-Re and acetyl-G-Rg_2_, respectively. The peaks **17**, **18**, **19**, **20**, **36**, **38**, and **47** were acetylated ginsenosides.

Peaks **1**, **59**, and **60** exhibited the fragment ions at *m*/*z* 455 corresponding to the oleanolic acid ([oleanolic acid − H]^−^) aglycone moiety, suggesting that they were the oleanolic acid-type ginsenosides. Peak **1** (*t*_R_ 21.51 min) showed a deprotonated molecular ion [M − H]^−^ at *m*/*z* 617.4066 which suggested the molecular formula was C_36_H_58_O_8_. In the MS^2^ spectrum, the fragment ions [M − H − Glc]^−^ at *m*/*z* 455.4067 could be attributed to the loss of Glc group. Finally, the peak **1** was assigned to be oleanolic acid-28-*O*-β-d-glucopyranosyl ester (OA-glc ester), a saponin isolated from the rhizomes of *Panax japonicus* var. *major* [29]. Peak **59** (*t*_R_ 99.43 min) showed a low abundant deprotonated molecular ion [M − H]^−^ at *m*/*z* 793.4344 which suggested the molecular formula was C_42_H_66_O_14_. In the MS^2^ spectrum, the fragment ions [M − H − Glc]^−^ at *m*/*z* 631.3751 could be attributed to the loss of Glc group, and the fragment ion [M − H − Glc − Glu A]^−^ at *m*/*z* 455.3527 could be attributed to the successive loss of Glc and glucosiduronyl (Glu A) groups. Finally, peak **59** was assigned to be chikusetsusaponin IVa, a compound detected and/or isolated in the rhizomes of *P. japonicus* var. *major* [30] and *P.* *stipuleanatus* [31]. Peak **60** (*t*_R_ 100.93 min) exhibited a deprotonated molecular ion [M − H]^−^ at *m*/*z* 925.4751 which suggested the molecular formula was C_47_H_74_O_18_. In the MS^2^ spectrum, the fragment ions [M − H − Glc]^−^ at *m*/*z* 763.4211, [M − H − Glc − Xyl]^−^ at *m*/*z* 631.3892, and [M − H − Glc − Xyl − Glu A]^−^ at *m*/*z* 455.3538 could be attributed to the successive loss of Glc, Xyl, and Glu A groups. Finally, the peak **60** was assigned to be pseudoginsenoside RT_1_ (pseudo-G-RT_1_), a saponin isolated in the rhizomes of *P.* *stipuleanatus* [31].

In this study, except for the ginsenosides characterized above, 2 polyacetylene glycosides were also tentatively identified, and TIC of Jilin GRR in negative ESI mode were shown in Figure 2. The details of identified 58 ginsenosides and two polyacetylene glycosides are summarized in Table S1.

### 2.2. Quantitative Analysis: Ginsenosides Determination

#### 2.2.1. Chromatographic Conditions

To achieve good separation of the chromatographic peaks in GRR, column types (Diamonsil™ ODS C_18_ (Beijing, China), Varian Microsorb TM-MV C_8_ (Walnut Creek, CA, USA), and Waters Symmetry^®^ ODS C_18_ columns (Waters Phillipsburg, NJ, USA), mobile phase compositions (methanol (MeOH)-water (H_2_O), acetonitrile (MeCN)-H_2_O, and formic acid aqueous solution), gradient elution procedure, and flow rate of the mobile phase (1.0, 0.8, 0.5 mL/min) were optimized, respectively. Finally, a Diamonsil™ ODS C_18_ column was used and the mobile phase consisted of (A) MeCN and (B) MeCN:H_2_O:0.1% phosphoric acid aqueous solution (5:90:8; *v*/*v*/*v*) with gradient elution. The flow rate was also changed alone with gradient elution (0–32 min, 0.8 mL/min; 32.1–110 min, 0.5 mL/min). Most of the analytes were successfully separated under the optimized condition, except **13** and **14**. In order to quantify **13** and **14**, the separation condition of these two ginsenosides was optimized individually.

#### 2.2.2. LC–DAD Method Validation

The proposed chromatographic method was validated. Good linearity was shown in Table 1. All the correlation coefficients were in the range of 0.9994–0.9999.

Under the established experimental conditions, the recoveries of these 14 ginsenosides in the three concentrations levels ranged from 95.93% to 103.93% and the RSD was 0.11%–2.64%. The results were shown in Table 2.

Intra- and inter-day precisions for these 14 ginsenosides yielded good results in the ranges of 0.16%–1.31% and 0.22%–1.24%, respectively.

**Figure 3:**
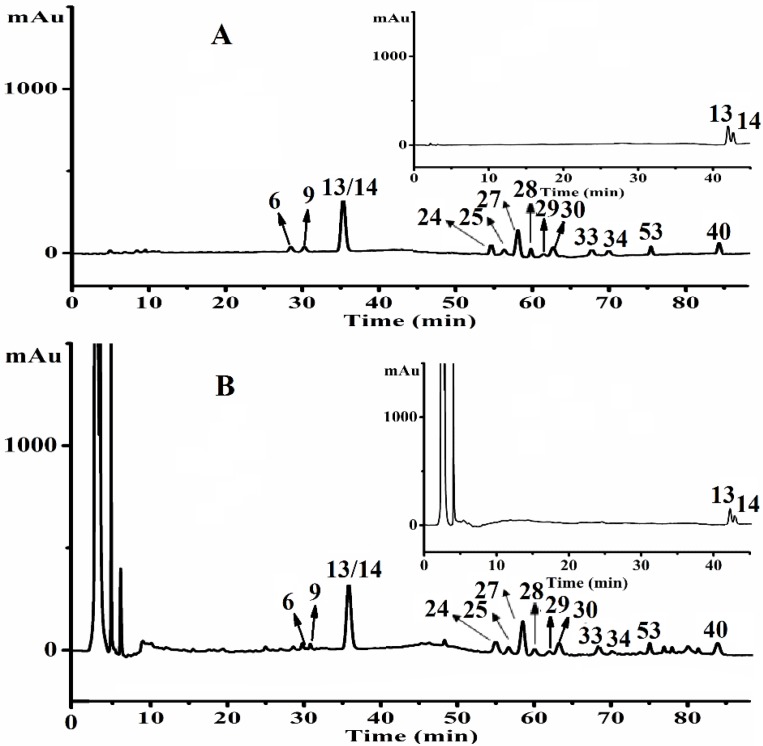
The LC–DAD chromatographic profiles of 14 reference standards (**6**: 20-glc-G-Rf; **9**: NG-R_1_; **13**: G-Rg_1_; **14**: G-Re; **24**: G-Rf; **25**: G-Ra_2_; **27**: G-Rb_1_; **28**: NG-R_2_; **29**: G-Ra_1_; **30**: G-Rc; **33**: G-Rb_2_; **34**: G-Rb_3_; **40**: G-Rd; **53**: G-Ro) (**A**); and samples of GRR (**B**).

As the main bioactive constituents in GRR, ginsenosides can be used as chemical markers for quality control purpose using chromatographic technique in combination with DAD detection. However, co-eluting the similarity of saponins within shorter retention time is difficult owing to the complexity of the GRR extracts. According to LC–DAD chromatography, 14 ginsenosides (showed in Figure 3, their chemical structures showed in Figure S1) were finally quantified to assess the quality of the GRR from different sources.

#### 2.2.3. HPLC Quantitative Analysis

The newly-developed method was subsequently applied to quantitative analyses of 14 ginsenosides in 66 samples collected from different locations (Table S2). Each sample was analyzed three times to determine the mean contents (shown in Table S3) and the content ranges (μg/g GRR) were 112–5326 for G-Ra_1_, 65–2656 for G-Ra_2_, 888–7723 for G-Rb_1_, 275–8247 for G-Rb_2_, 303–1609 for G-Rb_3_, 529–6122 for G-Rc, 387–4338 for G-Rd, 539–4815 for G-Re, 289–1577 for G-Rf, 870–6095 for G-Rg_1_, 667–6959 for G-Ro, 108–739 for 20-glc-G-Rf, 26–943 for NG-R_1_, and 25–699 for NG-R_2_. These results indicated that the contents of 14 ginsenosides varied greatly from the samples collected from different localities. The total contents of 14 ginsenosides were shown in Table S3. G-Ra_1_, Ra_2_, Rb_1_, Rb_2_, Rb_3_, Rc, and Rd represent the protopanaxadiol-type ginsenoside, whereas G-Re, Rf, Rg_1_, and 20-glc-G-Rf, NG-R_1_, as well as NG-R_2_ represent protopanaxatriol-type ginsenoside, and G-Ro represents oleanolic acid-type ginsenoside. These ginsenosides are considered as the biologically-active components of GRR [5]. The validated LC–DAD method is expected to provide the basis for the quality assessment of the GRR.

#### 2.2.4. Principal Component Analysis

**Figure 4:**
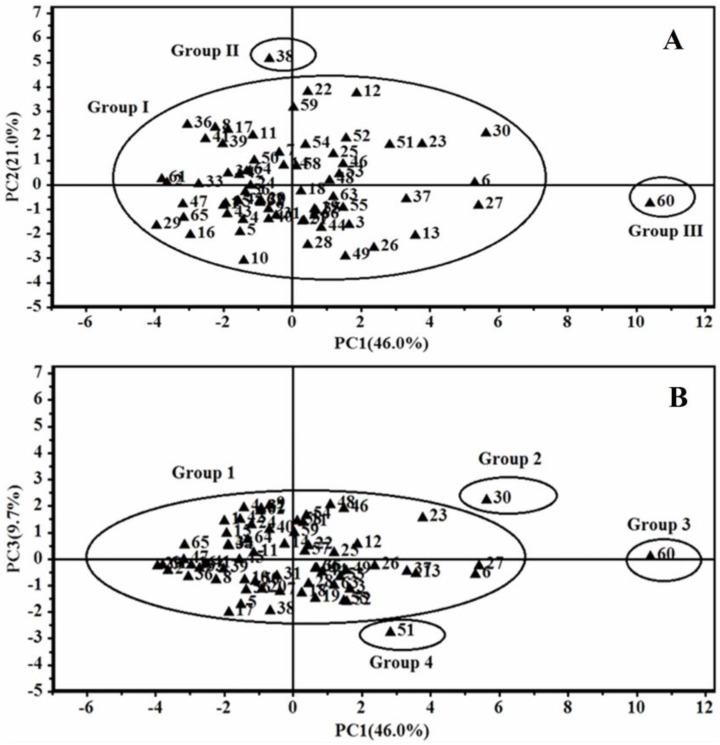
Scores plot of PCA for 66 samples of GRR. The scores plots for PC1 versus PC2 (**A**) and PC1 versus PC3 (**B**).

**Figure 5:**
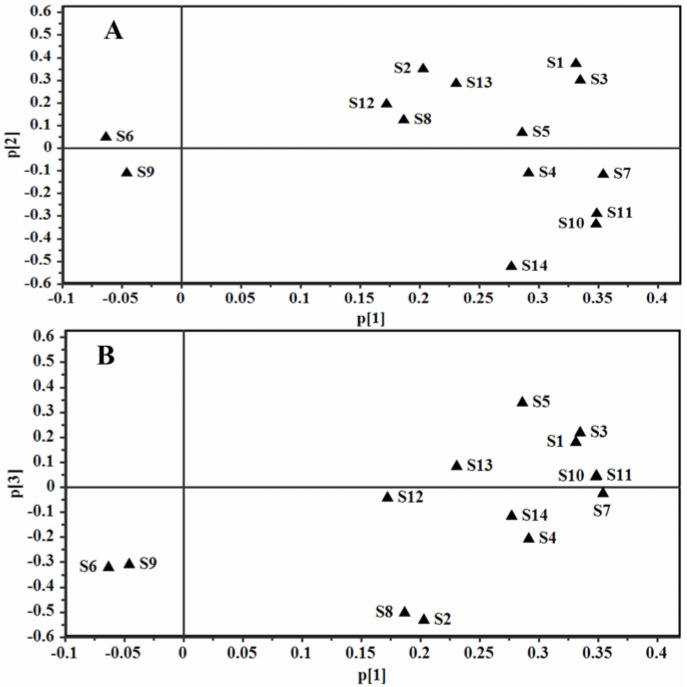
Loadings plot of PCA for 14 ginsenosides in their HPLC profiles of 66 samples of GRR (S1: 20-glc-G-Rf; S2: NG-R_1_; S3: G-Rg_1_; S4: G-Re; S5: G-Rf; S6: G-Ra_2_; S7: G-Rb_1_; S8: NG-R_2_; S9: G-Ra_1_; S10: G-Rc; S11: G-Rb_2_; S12: G-Rb_3_; S13: G-Ro; and S14: G-Rd). The loading plots for PC1 versus PC2 (**A**) and PC1 versus PC3 (**B**).

**Figure 6:**
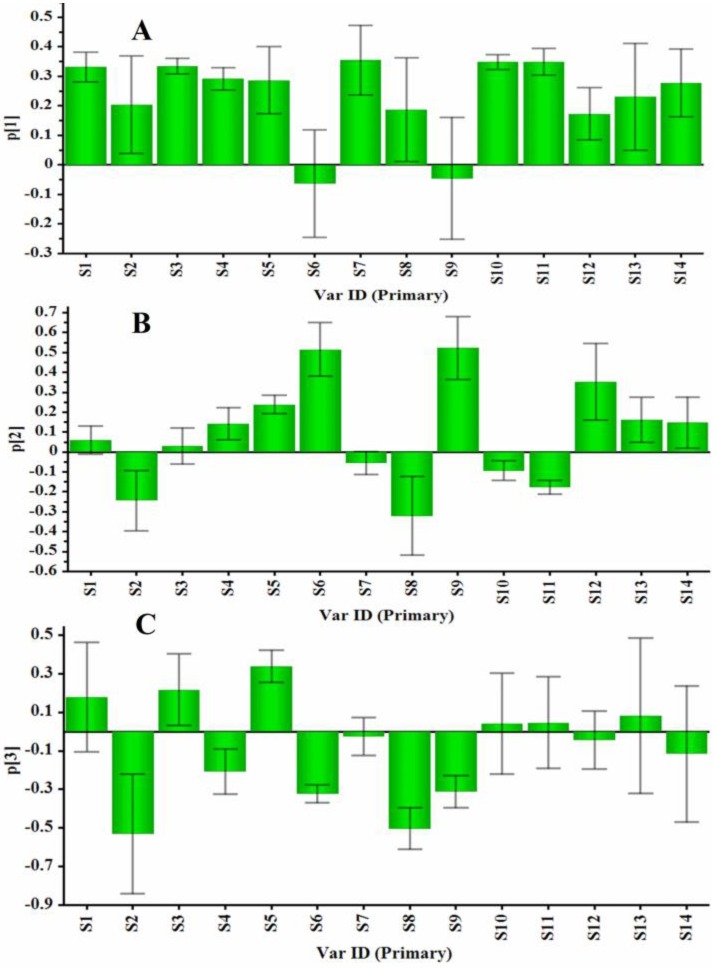
The influences of each variable on the first component (**A**), the second component (**B**), and the third component (**C**).

The LC–DAD contents of 14 ginsenosides were used for the evaluation of GRR collected from different regions. The contents of 14 ginsenosides were subjected to principal component analysis (PCA) and the results were shown in Figure 4. The first principal component 1 (PC1) contains the most variance in the data and the second principal component 2 (PC2) represents the maximum amount of variance not explained by PC1. The two ranking PCs, PC1 and PC2, described 46.0% and 21.0% of the total variability in the original observations, respectively, and they can accounted for 67.0% of the total variance. The scores plots for PC1 *versus* PC2 (Figure 4A) showed that 66 samples of GRR were classified into three groups (Groups I–III). Groups I (containing all samples except 38 and 60) and II (sample 38) were separated distinctly from Group III (sample 60) according to PC1. From Table S3, the total content of ginsenosides in sample 60 (48.62 mg/g) was much higher than those in the other samples. Group III was clustered by positive values of PC1, while Group I was clustered by positive and negative values of PC1. Group II was also clustered by negative values of PC1. Groups I and II were distinctly separated according to PC2 and Group II was clustered by positive values of PC2. The third principal component 3 (PC3) contains the remaining variance not explained by PC1 and PC2 by analogy and PC3 can describe 9.7% of the total variability in the original observations and consequently all the PCs accounts for 76.7% of the total variance. The score plots for PC1 *versus* PC3 (Figure 4B) also showed the ability to differentiate these 66 samples. Group 1, Group 2 and Group 4 were distinctly separated according to PC3, which were not separated in the scores plot for PC1 *versus* PC2. From Table S3, the total contents of ginsenosides in sample 30 (38.97 mg/g) and sample 51 (33.43 mg/g) were much higher than those of samples in Group 1 except sample 5 (34.07 m/g), sample 23 (34.88 mg/g) and sample 27 (36.55 mg/g). The loading plots for PC1 *versus* PC2 as well as PC1 *versus* PC3 were shown in Figure 5A,B. A more detailed interpretation of the loadings can be done from plots showing the loadings separately (shown in Figure 6). In Figure 6A–C, we can see the influence of each variable (S1~S14) on the first component, second component, and third component. Any ginsenoside have influence on the discrimination of the samples collected from different localities.

## 3. Experimental Section

### 3.1. Plant Materials

GRR (No. 1–66) were collected from different areas of Jilin and Heilongjiang provinces of China. The samples No. 1–62 were five-year old ginseng and No. 63–66 were four-year old ginseng. All samples were identified by Professors. Xiu-Wei Yang and Ying-Ping Wang who are a co-author of this paper, and all voucher specimens were deposited in the State Key Laboratory of Natural and Biomimetic Drugs, School of Pharmaceutical Sciences, Peking University (Beijing, China). GRR was dried and ground to powder that can pass through 40 meshes.

### 3.2. Chemical and Reagents

LC–MS grade MeCN and MeOH were obtained from J. T. Baker (Phillipsburg, NJ, USA). LC–grade MeCN, MeOH, and formic acid were got from Dikma Tech. Inc. (Beijing, China). H_2_O was gained from a Milli–Q Ultra-pure water system of our laboratory (Millipore, Billerica, MA, USA). Reference standards of ginsenosides (G)-Ra_1_ (**29**), Ra_2_ (**25**), Ra_3_ (**26**), Rb_1_ (**27**), Rb_2_ (**33**), Rb_3_ (**34**), Rc (**30**), Rd (**40**), Re (**14**), Re_1_ (**8**), Re_2_ (**10**), Re_3_ (**3**), Re_4_ (**5**), Rf (**24**), Rg_1_ (**13**), Rg_2_ (**31**), Rg_3_ (**58**), Ro (**53**), Rs_1_ (**43**), Rs_2_ (**41**), 20-*O*-glucopyranosylginsenoside Rf (20-glc-G-Rf, **6**), ginsenoside Ro methyl ester (G-RoMe, **50**), notoginsenosides (NG)-N (**12**), R_1_ (**9**), R_2_ (**28**), R_4_ (**21**), quinquenoside (PQ)-R_1_ (**37**), and koryoginsenoside (KG)-R_1_ (**22**) were isolated from GRR [32,33] and Rh_1_ (**32**) was isolated from hydrolysate of total saponins in the stems-leaves of *P. ginseng* [17] in our previous research. Oleanolic acid (**4**), malonyl-ginsenoside (Ma-G)-Rb_2_ (**44**), and chikusetsusaponin IV (**56**) were supplied by Natural Product Sample Library in State Key Laboratory of Natural and Biomimetic Drugs of Peking University. Their chemical structures (showed in Figure S1) were determined by MS and 2D NMR spectra. Purities of all the reference standards were above 99.0% determined with LC–DAD method.

### 3.3. Chromatographic and Mass Spectrometric Conditions

Qualitative analysis was performed on a Shimadzu LC system (equipped with a binary LC-20AD pump, a CBM-20A system controller, a SPD-M20A PDA detector, an SIL-20AC autosampler, and a CTO–20A column oven) coupled to an ESI-IT-TOF mass spectrometer (Shimadzu, Kyoto, Japan), and the data analysis was performed on a Shimadzu software (Shimadzu LCMS solution Version 3.60, Formula Predictor Version 1.2, and Accurate Mass Calculator). Quantitative determination was performed on an Agilent 1260 LC system equipped with a quaternary pump, diode-array detector, a column oven, and an autosampler.

All qualitative and quantitative separation was successfully achieved on a Diamonsil™ ODS C_18_ column (250 × 4.6 mm i.d., 5 μm). The mobile phases for the LC-UV method consisted of (A) MeCN and (B) MeCN–H_2_O–0.1% phosphoric acid aqueous solution (5:90:8; *v*/*v*/*v*), and for the LC-MS method consisted of (A) MeCN and (B) MeCN–H_2_O–0.1% formic acid aqueous solution (5:90:8; *v*/*v*/*v*). The optimized gradient elution was described as follows: 0–20 min, 10%–20% A; 20–30 min, 20%–22% A; 30–40 min, 22%–31% A; 40–75 min, 31%–33% A; 75–80 min, 33%–40% A; 80–90 min, 40%–50% A; 90–100 min, 50%–60% A; 100–110 min, 60%–70% A. The flow rate was also with the gradient (0–32 min, 0.8 mL/min; 32.1–110 min, 0.5 mL/min). The inject volume was 10 μL and the column temperature was set at 35 °C. The detection wavelength was set at 203 nm. The separation condition for ginsenosides **13** and **14** was optimized as: (A) MeCN and (B) H_2_O with gradient elution (0–30 min, 20% A; and 30–45 min, 20%–30% A). During qualitative analysis, the ESI source was in negative ion mode and the selection of precursor ions for MS^2^ and MS^3^ were set as “automatic” in LCMS solution software (Shimadzu, Kyoto, Japan), which means the selection was data-dependent. The base peak chromatogram (BPC) intensity threshold was used, and the execution trigger was set as “start level at 10,000 (BPC intensity), stop level at 95% of start level”. The dynamic exclusion was also used: the period was set to be 10 s, and the list size was 1480 for MS^2^ and 20 for MS^3^. The mass spectrometer was in full-scan ranges of *m*/*z* 500–1500 for MS^1^ and *m*/*z* 50–1000 for MS^2^ and MS^3^. The temperature of heat block and curved desolvation lines were all set at 200 °C. The diversion ratio was 1:4 and the flow of desolvation gas (N_2_) was 1.5 L/min.

### 3.4. Preparation of Sample and Standard Solutions

The reference standards of **6**, **9**, **13**, **14**, **24**, **25**, **27**, **28**, **29**, **30**, **33**, **34**, **40**, **53** were dissolved with MeOH and stocked at 4 °C, respectively. The reference standards were divided into two groups. The first group included **6**, **9**, **24**, **25**, **27**, **28**, **29**, **30**, **33**, **34**, **40**, and **53**, while the second group included **13** and **14**. Specific amount of these reference compound stock solutions were mixed to obtain the mixed reference standard solution. The final concentrations in one milliliter of MeOH in the first group were 3000 μg of **6**, 3000 μg of **9**, 12,000 μg of **24**, 6000 μg of **25**, 40,000 μg of **27**, 3000 μg of **28**, 9000 μg of **29**, 20,000 μg of **30**, 20,000 μg of **33**, 6000 μg of **34**, 24,000 μg of **40**, and 24,000 μg of **53**. The final concentrations in one milliliter of MeOH in the second group were 20,000 μg of **13** and 15,000 μg of **14**.

The first group of mixed reference standard solution was then diluted step by step with MeOH to obtain a series of standard solutions, and the concentrations of each reference standard were **6**: 240, 120, 60, 30, 15, 7.5 μg/mL; **9**: 240, 120, 60, 30, 15, 7.5 μg/mL; **24**: 960, 480, 240, 120, 60, 30 μg/mL; **25**: 480, 240, 120, 60, 30, 15 μg/mL; **27**: 3200, 1600, 800, 400, 200, 100 μg/mL; **28**: 240, 120, 60, 30, 15, 7.5 μg/mL; **29**: 720, 360, 180, 90, 45, 22.5 μg/mL; **30**: 1600, 800, 400, 200, 100, 50 μg/mL; **33**: 1600, 800, 400, 200, 100, 50 μg/mL; **34**: 480, 240, 120, 60, 30, 15 μg/mL; **40**: 1920, 960, 480, 240, 120, 60 μg/mL; **53**: 1920, 960, 480, 240, 60, and 7.5 μg/mL. The second group of mixed reference standard solution was also diluted step by step with MeOH to obtain a series of standard solutions, and the concentrations of each reference standard were **13**: 1600, 800, 400, 200, 100, 50 μg/mL; **14**: 1200, 600, 300, 150, 75, and 37.5 μg/mL. The solutions were filtered using a 0.45 μm filter membrane prior to quantitative analysis or qualitative analysis.

All analysis samples were treated according to the previous report [34]. The powdered GRR (40 mesh size) was submitted to ultrasound-assisted solvent extraction: 1.0 g of the sample was extracted three times, each time for 30 min, with 20 mL of 70% aqueous MeOH using a sonicator at 40 kHz and 250 W at 25 °C. Filtered extracted solutions were combined and evaporated to dryness using a rotatory evaporator at 40 °C. The residue was then dissolved in 5 mL of 70% aqueous MeOH and filtered through a 0.45 μm filter membrane prior to analysis.

### 3.5. LC–DAD Method Validation

The linearity, lower limits of detection (LLOD), and quantification (LLOQ), intra- and inter-day precisions, as well as the accuracy were evaluated to validate the proposed LC–DAD method.

The LC analysis of the standard solutions of these 14 ginsenosides gave the calibration curves, from which the linearity was determined. In this experiment, five increments of concentrations for the 14 ginsenosides were injected in triplicate, and the calibration curves were established by determining the peak areas against the concentration of each analyte. The correlation coefficients were adopted to verify the linearity of the calibration curves. Intra- and inter-day variations were used to determine the precision of the developed method. A 1.0 g aliquot of GRR powder was extracted and analyzed as described in Section 3.4. The intra- and inter-day precisions were performed by injecting the samples six times on one day and three successive days, respectively. The results were expressed with the relative standard deviations (RSD). The recovery test was used to evaluate the accuracy of this quantification method. Accurate amounts of the 14 ginsenosides were added to 1.0 g of GRR powder and then extracted and analyzed as described in Section 3.3 and Section 3.4. Each sample was injected three times and the average recovery was then calculated. LLOD and LLOQ were defined as signal-to-noise ratios of 3:1 and 10:1, respectively. The standard solutions of the 14 ginsenosides for LLOD and LLOQ were prepared by sequential dilution.

## 4. Conclusions

A new and reliable method for comprehensive chemical analysis of the GRR by LC–ESI-IT-TOF-MS^n^ combined with chemometrics was developed to evaluate the quality of GRR and the developed LC–ESI-IT-TOF-MS^n^ method has the potential to be applied for ginsenosides detection in the remaining 66 extracts. Finally, 58 ginsenosides in the GRR extract were unequivocally identified or tentatively assigned. Chemometrics were successfully applied to comprehensive chemical analysis of the GRR samples in 66 producing areas to explain the difference. The results indicated that the contents of 14 investigated ginsenosides varied greatly among the samples collected from different localities. Moreover, the contents of G-Rb_1_, Rb_2_, Rc, Rd, Re, Rf, Rg_1_, and Ro were found to be much higher than other ginsenosides and the total contents of these eight ginsenosides were mostly above 80%, even can up to 95.5%. These eight ginsenosides should be selected as markers to evaluate the quality of GRR samples. The developed method provided a potential analytical platform for quality control of GRR and also should be useful to evaluate the quality of GRR related products. This will play a role to provide a basis for the potential study of pharmacological effects.

## Figures and Tables

**Table 1:** Linear calibration curves of 14 ginsenosides.

Analyte	Calibration Curve	*r*^2^	Linear Range (μg/mL)	LLOD (ng)	LLOQ (ng)
G-Ra_1_	*y* = 3.625*x* + 37.274	0.9995	22.5–720	5.78	19.23
G-Ra_2_	*y* = 3.507*x* + 7.198	0.9999	15–480	2.94	9.79
G-Rb_1_	*y* = 5.455*x* + 193.800	0.9995	100–3200	3.31	11.04
G-Rb_2_	*y* = 4.634*x* + 93.950	0.9995	50–1600	6.09	20.30
G-Rb_3_	*y* = 4.288*x* + 2.264	0.9996	15–480	1.83	6.09
G-Rc	*y* = 5.289*x* + 97.009	0.9996	50–1600	3.96	13.19
G-Rd	*y* = 3.707*x* + 132.820	0.9998	60–1920	6.40	21.33
G-Re	*y* = 3.694*x* + 73.629	0.9996	37.5–1200	7.74	25.81
G-Rf	*y =* 7.637*x +* 136.930	0.9995	30–960	3.43	11.44
G-Rg_1_	*y* = 4.202*x* + 89.410	0.9999	50–1600	6.90	23.00
G-Ro	*y* = 3.873*x* + 42.919	0.9996	7.5–1920	8.82	29.39
20-glc-G-Rf	*y* = 3.446*x* + 18.670	0.9996	7.5–240	0.73	2.44
NG-R_1_	*y* = 3.359*x* + 19.016	0.9994	7.5–240	0.66	2.21
NG-R_2_	*y* = 7.407*x* + 20.821	0.9998	7.5–480	1.97	6.57

**Table 2:** The recoveries of 14 ginsenosides.

Analyte	Original Amount (μg)	Spiked Amount (μg)	Total Amount Detected (μg)	Mean Recovery (%)	RSD (%)
G-Ra_1_	152.99	225	377 ± 2	99.72	0.46
		280	333 ± 2	100.11	0.72
		135	286 ± 2	98.52	0.70
G-Ra_2_	82.45	120	202 ± 1	99.74	0.69
		90	173 ± 1	100.43	0.52
		60	144 ± 1	102.03	0.69
G-Rb_1_	214.26	240	455 ± 1	100.20	0.22
		200	413 ± 2	99.49	0.53
		160	377 ± 1	101.26	0.25
G-Rb_2_	115.45	140	257 ± 2	101.39	0.94
		120	238 ± 3	102.40	1.30
		100	218 ± 2	102.73	1.03
G-Rb_3_	76.30	120	194 ± 2	98.32	1.22
		90	165 ± 3	98.32	1.67
		60	137 ± 4	101.67	2.64
G-Rc	114.91	140	254 ± 1	99.16	0.33
		120	235 ± 0	99.80	0.19
		100	215 ± 0	100.17	0.11
G-Rd	71.07	84	158 ± 2	103.63	1.54
		72	142 ± 2	98.37	1.44
		60	134 ± 3	104.40	2.14
G-Re	196.86	180	376 ± 0	99.37	0.11
		150	345 ± 2	98.55	0.56
		120	317 ± 1	100.31	0.28
G-Rf	71.69	84	154 ± 2	97.41	1.14
		72	143 ± 2	99.63	1.14
		60	129 ± 0	95.37	0.27
G-Rg_1_	240.78	300	546 ± 1	101.76	0.20
		240	487 ± 2	102.44	0.34
		180	428 ± 1	103.93	0.15
G-Ro	128.41	144	271 ± 3	99.27	1.19
		120	246 ± 5	97.67	1.96
		96	222 ± 4	97.90	1.84
20-glc-G-Rf	29.65	45	73.7 ± 1	97.80	1.60
		30	60.7 ± 0.9	103.43	1.46
		15	45.0 ± 0.3	102.60	0.58
NG-R_1_	18.55	24	41.7 ± 0.3	96.65	0.71
		18	36.7 ± 0.2	100.81	0.52
		15	34.0 ± 0.3	103.22	0.99
NG-R_2_	11.99	18	29.3 ± 0.3	95.93	1.15
		12	24.1 ± 0.5	100.92	2.22
		6	17.9 ± 0.4	98.90	1.97

## Supplementary Materials

The LC–ESI-IT-TOF-MS^n^ data of saponins/polyacetylenes in GRR, list of production areas of GRR, the content of ginsenosides in 66 producing areas, and chemical structures of ginsenosides are available as Supplementary Materials, which may be accessed at: http://www.mdpi.com/1420-3049/21/5/603/s1.
