# Supplementary material for: High-Performance Liquid Chromatography with Diode Array Detector and Electrospray Ionization Ion Trap Time-of-Flight Tandem Mass Spectrometry to Evaluate Ginseng Roots and Rhizomes from Different Regions
Source: Molecules. 2016 May 9;21(5):603. doi: 10.3390/molecules21050603 (PMC6274567; doi:10.3390/molecules21050603)
Supplement: Supplementary file 1 [file molecules-21-00603-s001.pdf]

Supplementary Materials: High Performance Liquid Chromatography with Diode Array Detector and Electrospray Ionization Ion Trap Time-of-Flight Tandem Mass Spectrometry to Evaluate Ginseng Roots and Rhizomes from Different Regions

Hong-Ping Wang, You-Bo Zhang, Xiu-Wei Yang, Xin-Bao Yang, Wei Xu, Feng Xu, Shao-Qing Cai, Ying-Ping Wang, Yong-Hua Xu and Lian-Xue Zhang

Table S1. The LC–ESI-IT-TOF-MS^n data of saponins/polyacetylenes in GRR.

No.	t_R (min)	Compounds	Molecular Formula	Measured Value ^{a} (*m*/*z*)	Diff (ppm)	MS^n
1	21.51	OA-glc ester	$C_{36}H_{58}O_8$	617.4066 $^{\#\#}$	0.21	455.4067 [M − H − Glc]$^-$
2	25.09	20-glc-G-Rf isomer	$C_{48}H_{82}O_{19}$	1007.5751 $^{\#}$	3.21	961.5399 [M − H]$^-$, 799.4883 [M − H − Glc]$^-$, 637.4323 [M − H − 2Glc]$^-$, 475.3779 [M − H − 3Glc]$^-$
3	26.34	G-Re$_3$	$C_{48}H_{82}O_{19}$	1007.5405 $^{\#}$	−0.22	961.5291 [M − H]$^-$, 799.4764 [M − H − Glc]$^-$, 637.44291 [M − H − 2Glc]$^-$, 475.43741M − H − 3Glc]$^-$
4	27.81	oleanolic acid	$C_{30}H_{48}O_3$	455.3529 $^{\#\#}$	0.09	455.3567 [M − H]$^-$
5	28.02	G-Re$_4$	$C_{47}H_{80}O_{18}$	977.5224 $^{\#}$	−0.99	931.4966 [M − H]$^-$, 799.3831 [M − H − Ara(f)]$^-$, 637.4328 [M − H − Ara(f) − Glc]$^-$ 475.3898 [M − H − Ara(f) − 2Glc]$^-$
6	28.32	20-glc-G-Rf	$C_{48}H_{82}O_{19}$	1007.5300 $^{\#}$	−1.26	961.5296 [M − H]$^-$, 799.4779 [M − H − Glc]$^-$, 637.4230 [M − H − 2Glc]$^-$, 475.3733 [M − H − 3Glc]$^-$
7	29.15	G-Re$_4$ isomer	$C_{47}H_{80}O_{18}$	977.5145 $^{\#}$	−1.80	931.5087 [M − H]$^-$, 799.3844 [M − H − Ara(f)]$^-$, 637.4358 [M − H − Ara − Glc]$^-$, 475.3815 [M − H − Ara − 2Glc]$^-$
8	29.64	G-Re$_1$	$C_{48}H_{82}O_{19}$	961.5302 $^{\#\#}$	−0.73	799.4793 [M − H − Glc]$^-$, 637.4293 [M − H − 2Glc]$^-$, 475.3753 [M − H − 3Glc]$^-$
9	30.40	NG-R$_1$	$C_{47}H_{80}O_{18}$	977.5201 $^{\#}$	−1.23	931.5139 [M − H]$^-$, 799.4808 [M − H − Xyl]$^-$, 637.4312 [M − H − Xyl − Glc]$^-$, 475.3707 [M − H − Xyl − 2Glc]$^-$
10	30.89	G-Re$_2$	$C_{48}H_{82}O_{19}$	1007.5431 $^{\#}$	0.04	961.5316 [M − H]$^-$, 799.4809 [M − H − Glc]$^-$, 637.4264 [M − H − 2Glc]$^-$, 475.3760 [M − H − 3Glc]$^-$
11	31.90	8(*E*)-decene-4,6-diyne-1-*O*-β-D-glucopyranosyl-(1→2)-*O*-β-D-glucopyranoside	$C_{22}H_{32}O_{11}$	517.1958 $^{\#}$	0.72	471.1855 [M − H]$^-$
12	32.38	NG-N	$C_{48}H_{82}O_{19}$	1007.5420 $^{\#}$	−0.07	961.5340 [M − H]$^-$, 799.4915 [M − H − Glc]$^-$, 637.4263 [M − H − 2Glc]$^-$, 475.3795 [M − H − 3Glc]$^-$
13	35.40	G-Rg$_1$	$C_{42}H_{72}O_{14}$	845.4853 $^{\#}$	−0.54	799.4840 [M − H]$^-$, 637.4308 [M − H − Glc]$^-$, 475.3776 [M − H − 2Glc]$^-$
14	35.85	G-Re	$C_{48}H_{82}O_{18}$	991.5399 $^{\#}$	−0.80	945.5271 [M − H]$^-$, 783.4811 [M − H − Glc]$^-$, 637.4235 [M − H − Glc − Rha]$^-$, 475.3739 [M − H − 2Glc − Rha]$^-$
15	46.02	G-F$_3$/G-F$_5$	$C_{41}H_{70}O_{13}$	815.4762 $^{\#}$	−0.38	769.4717 [M − H]$^-$, 637.4193 [M − H − Ara]$^-$, 475.3685 [M − H − Ara − Glc]$^-$
16	46.76	G-F$_5$/G-F$_3$	$C_{41}H_{70}O_{13}$	815.4739 $^{\#}$	−0.66	769.4708 [M − H]$^-$, 637.4251 [M − H − Ara]$^-$, 475.3767 [M − H − Ara − Glc]$^-$
17	47.10	acetyl-G-Rg$_1$/isomer	$C_{44}H_{74}O_{15}$	887.4947 $^{\#}$	−0.64	841.4784 [M − H]$^-$, 799.4776 [M − H − Ac]$^-$, 637.4241 [M − H − Ac − Glc]$^-$, 475.3706 [M − H − Ac − 2Glc]$^-$
18	48.67	acetyl-G-Rg$_1$/isomer	$C_{44}H_{74}O_{15}$	887.4976 $^{\#}$	−0.32	841.4880 [M − H]$^-$, 799.4822 [M − H − Ac]$^-$, 637.4168 [M − H − Ac − Glc]$^-$, 475.3693 [M − H − Ac − 2Glc]$^-$
19	49.47	acetyl-G-Re	$C_{49}H_{80}O_{20}$	1033.5503 $^{\#}$	2.75	987.5360 [M − H]$^-$, 945.5391 [M − H − Ac]$^-$, 799.4674 [M − H − Ac − Rha]$^-$, 637.4262 [M − H − Ac − Rha − Glc]$^-$, 475.3693 [M − H − Ac − Rha − 2Glc]$^-$
20	50.25	acetyl-G-Rg$_1$/isomer	$C_{44}H_{74}O_{15}$	887.4948 $^{\#}$	−0.63	841.4827 [M − H]$^-$, 799.4719 [M − H − Ac]$^-$, 637.4210 [M − H − Ac − Glc]$^-$, 475.3750 [M − H − Ac − 2Glc]$^-$
21	50.87	NG-R$_4$	$C_{55}H_{100}O_{30}$	1239.6276 $^{\#}$	0.44	945.5311 [M − H − Xyl − Glc]$^-$, 783.4828 [M − H − Xyl − 2Glc]$^-$, 621.3040 [M − H − Xyl − 3Glc]$^-$, 459.3800 [M − H − Xyl − 4Glc]$^-$
22	53.06	KG-R$_1$	$C_{46}H_{76}O_{15}$	913.5036 $^{\#}$	−1.37	867.5025 [M − H]$^-$, 799.4770 [M − H − but − 2-enoyl]$^-$, 637.4192 [M − H − but − 2-enoyl − Glc]$^-$, 475.3770 [M −

						H − but − 2-enoyl − 2Glc]$^-$
23	53.44	8(*E*)-decene-4,6-diyne-1-*O*-β-D-glucopyranoside	$C_{16}H_{22}O_6$	355.1358 [#]	−0.99	309.1351 [M − H]$^-$
24	54.95	G-Rf	$C_{42}H_{72}O_{14}$	799.4796 [##]	−0.60	637.4235 [M − H − Glc]$^-$, 475.3741 [M − H − 2Glc]$^-$
25	56.45	G-Ra_2	$C_{58}H_{98}O_{26}$	1209.6092 [##]	−1.45	1077.5672 [M − H − Xyl]$^-$, 945.5271 [M − H − Xyl − Ara(f)]$^-$, 783.4794 [M − H − Xyl − Ara(f) − Glc]$^-$, 621.4279 [M − H − Xyl − Ara(f) − 2Glc]$^-$, 459.3763 [M − H − Xyl − Ara(f) − 3Glc]$^-$
26	57.54	G-Ra_3	$C_{59}H_{100}O_{27}$	1239.6247 [##]	−1.02	1077.5787 [M − H − Xyl]$^-$, 945.5294 [M − H − Xyl − Glc]$^-$, 783.4817 [M − H − Xyl − 2Glc]$^-$, 621.4311 [M − H − Xyl − 3Glc]$^-$, 459.3760 [M − H − Xyl − 4Glc]$^-$
27	58.45	G-Rb_1	$C_{54}H_{92}O_{23}$	1107.5831 [##]	−1.08	945.5301 [M − H − Glc]$^-$, 783.4800 [M − H − 2Glc]$^-$, 621.4293 [M − H − 3Glc]$^-$, 459.3783 [M − H − 4Glc]$^-$
28	59.75	NG-R_2	$C_{41}H_{70}O_{13}$	815.4747 [#]	−0.56	769.4754 [M − H]$^-$, 637.4298 [M − H − Xyl]$^-$, 475.3733 [M − H − Xyl − Glc]$^-$
29	62.36	G-Ra_1	$C_{58}H_{98}O_{26}$	1209.6261 [##]	−0.06	1077.5694 [M − H − Xyl]$^-$, 945.5309 [M − H − Xyl − Ara(p)]$^-$, 783.4789 [M − H − Xyl − Ara(p) − Glc]$^-$, 621.4299 [M − H − Xyl − Ara(p) − 2Glc]$^-$, 459.3748 [M − H − Xyl − Ara(p) − 3Glc]$^-$
30	64.00	G-Rc	$C_{53}H_{90}O_{22}$	1077.5786 [##]	−0.55	945.5252 [M − H − Ara(f)]$^-$, 783.4817 [M − H − Ara(f) − Glc]$^-$, 621.4293 [M − H − Ara(f) − 2Glc]$^-$, 459.3789 [M − H − Xyl − Ara(f) − 3Glc]$^-$
31	65.22	G-Rg_2	$C_{42}H_{72}O_{13}$	829.4996 [#]	0.57	783.4785 [M − H]$^-$, 475.3708 [M − H − Rha − Glc]$^-$
32	67.95	G-Rh_1	$C_{36}H_{62}O_9$	683.4354 [#]	−0.23	637.4204 [M − H]$^-$, 475.3731 [M − H − Glc]$^-$
33	68.99	G-Rb_2	$C_{53}H_{90}O_{22}$	1123.5814 [#]	−0.77	1077.5706 [M − H]$^-$, 945.5313 [M − H − Ara(p)]$^-$, 783.4810 [M − H − Ara(p) − Glc]$^-$, 621.4307 [M − H − Ara(p) − 2Glc]$^-$, 459.3789 [M − H − Ara(p) − 3Glc]$^-$
34	71.35	G-Rb_3	$C_{53}H_{90}O_{22}$	1077.5803 [##]	−0.39	945.5221 [M − H − Xyl]$^-$, 783.4786 [M − H − Xyl − Glc]$^-$, 621.4301 [M − H − Xyl − 2Glc]$^-$, 459.3769 [M − H − Xyl − 3Glc]$^-$
35	72.61	PQ-R_1 isomer	$C_{56}H_{94}O_{24}$	1149.6154 [##]	0.84	1107.5198 [M − H − Ac]$^-$, 945.5260 [M − H − Ac − Glc]$^-$, 783.4910 [M − H − Ac − 2Glc]$^-$, 621.4290 [M − H − Ac − 3Glc]$^-$, 459.3816 [M − H − Ac − 4Glc]$^-$
36	75.85	yesanchinoside D	$C_{44}H_{74}O_{15}$	887.4952 [#]	−0.59	841.4930 [M − H]$^-$, 799.4742 [M − H − Ac]$^-$, 637.4177 [M − H − Ac − Glc]$^-$, 475.3693 [M − H − Ac − 2Glc]$^-$,
37	77.20	PQ-R_1	$C_{56}H_{94}O_{24}$	1149.5971 [##]	−0.75	1107.5799 [M − H − Ac]$^-$, 945.5265 [M − H − Ac − Glc]$^-$, 783.4806 [M − H − Ac − 2Glc]$^-$, 621.4293 [M − H − Ac − 3Glc]$^-$, 459.3755 [M − H − Ac − 4Glc]$^-$
38	79.84	yesanchinoside D/isomer	$C_{44}H_{74}O_{15}$	887.5109 [#]	1.18	841.4865 [M − H]$^-$, 799.4733 [M − H − Ac]$^-$, 637.4186 [M − H − Ac − Glc]$^-$, 475.3740 [M − H − Ac − 2Glc]$^-$
39	82.99	Ma-G-Rb_1 isomer	$C_{57}H_{94}O_{26}$	1193.5859 [##]	−0.80	1149.6016 [M − H − CO_2]$^-$, 945.5296 [M − H − CO_2 − Ac − Glc]$^-$, 783.4710 [M − H − CO_2 − Ac − 2Glc]$^-$, 621.4353 [M − H − CO_2 − Ac − 3Glc]$^-$, 459.3823 [M − H − CO_2 − Ac − 4Glc]$^-$
40	83.83	G-Rd	$C_{48}H_{82}O_{18}$	991.5432 [#]	−0.46	945.5425 [M − H]$^-$, 783.4902 [M − H − Glc]$^-$, 621.4373 [M − H − 2Glc]$^-$, 459.3833 [M − H − 3Glc]$^-$
41	84.29	G-Rs_2	$C_{55}H_{92}O_{23}$	1165.5913 [#]	−0.80	1119.5891 [M − H]$^-$, 783.4788 [M − H − Ac − Ara(f) − Glc]$^-$, 621.4314 [M − H − Ac − Ara(f) − 2Glc]$^-$, 459.3755 [M − H − Ac − Ara(f) − 3Glc]$^-$
42	85.39	Ma-G-Rb_1	$C_{57}H_{94}O_{26}$	1193.5906 [##]	−0.41	1149.5951 [M − H − CO_2]$^-$, 945.5169 [M − H − CO_2 − Ac − Glc]$^-$, 783.4901 [M − H − CO_2 − Ac − 2Glc]$^-$, 621.4359 [M − H − CO_2 − Ac − 3Glc]$^-$, 459.3828 [M − H − CO_2 − Ac − 4Glc]$^-$
43	86.45	G-Rs_1	$C_{55}H_{92}O_{23}$	1165.5817 [#]	−1.62	1119.5851 [M − H]$^-$, 621.3009 [M − H − Ac − Ara(p) − 2Glc]$^-$, 459.3835 [M − H − Ac − Ara(p) − 3Glc]$^-$
44	87.00	Ma-G-Rb_2	$C_{56}H_{92}O_{25}$	1163.5711 [##]	−1.19	1119.5811 [M − H − CO_2]$^-$, 1077.5677 [M − H − CO_2 − Ac]$^-$, 945.5196 [M − H − CO_2 − Ac − Ara(p)]$^-$, 783.4834 [M − H − CO_2 − Ac − Ara(p) − Glc]$^-$, 621.4347 [M − H − CO_2 − Ac − Ara(p) − 2Glc]$^-$, 459.3818 [M − H − CO_2 − Ac − Ara(p) − 3Glc]$^-$

45	87.35	Ma-G-Rb$_3$	$C_{56}H_{92}O_{25}$	1163.5835 ##	−0.12	1119.5815 [M − H − CO$_2$]$^-$, 1077.5680 [M − H − CO$_2$ − Ac]$^-$, 945.5384 [M − H − CO$_2$ − Ac − Xyl]$^-$, 783.4683 [M − H − CO$_2$ − Ac − Xyl − Glc]$^-$, 621.4349 [M − H − CO$_2$ − Ac − Xyl − 2Glc]$^-$, 459.3830 [M − H − CO$_2$ − Ac − Xyl − 3Glc]$^-$
46	87.42	G-Rd isomer	$C_{48}H_{82}O_{18}$	991.5412 #	−0.67	945.5493 [M − H]$^-$, 783.4929 [M − H − Glc]$^-$, 621.4438 [M − H − 2Glc]$^-$, 459.3833 [M − H − 3Glc]$^-$
47	87.82	acetyl-G-Rg$_2$	$C_{44}H_{74}O_{14}$	825.4974 ##	−0.31	783.4869 [M − H − Ac]$^-$, 637.4307 [M − H − Ac − Rha]$^-$, 475.3736 [M − H − Ac − Rha − Glc]$^-$
48	89.00	Ma-G-Rc	$C_{56}H_{92}O_{25}$	1163.5729 ##	−1.03	1119.5873 [M − H − CO$_2$]$^-$, 1077.5718 [M − H − CO$_2$ − Ac]$^-$, 945.5366 [M − H − CO$_2$ − Ac − Ara(f)]$^-$, 783.4806 [M − H − CO$_2$ − Ac − Ara(f) − Glc]$^-$, 621.4457 [M − H − CO$_2$ − Ac − Ara(f) − 2Glc]$^-$, 459.3828 [M − H − CO$_2$ − Ac − Ara(f) − 3Glc]$^-$
49	89.85	Ma-G-Rc isomer	$C_{56}H_{92}O_{25}$	1163.5953 ##	−1.03	1119.5559 [M − H − CO$_2$]$^-$, 1077.5670 [M − H − CO$_2$ − Ac]$^-$, 945.5191 [M − H − CO$_2$ − Ac − Ara(p)]$^-$, 783.4845 [M − H − CO$_2$ − AcAra(p) − Glc]$^-$, 621.4352 [M − H − CO$_2$ − Ac − Ara(p) − 2Glc]$^-$, 459.3825 [M − H − CO$_2$ − Ac − Ara(p) − 3Glc]$^-$
50	90.30	G-RoMe	$C_{49}H_{78}O_{19}$	1015.5079 #	−0.34	969.5010 [M − H]$^-$, 807.4505 [M − H − Glc]$^-$, 455.3522 [M − H − 2Glc − Glu A]$^-$
51	90.71	VG-R$_{16}$	$C_{47}H_{80}O_{17}$	961.5356 #	−0.17	915.5216 [M − H]$^-$, 783.4865 [M − H − Xyl]$^-$, 621.4305 [M − H − Xyl − Glc]$^-$, 459.3789 [M − H − Xyl − 2Glc]$^-$
52	90.83	pseudo-G-RC$_1$	$C_{50}H_{84}O_{19}$	1033.5592 #	0.09	987.5461 [M − H]$^-$, 945.5289 [M − H − Ac]$^-$, 783.4817 [M − H − Ac − Glc]$^-$, 621.4320 [M − H − Ac − 2Glc]$^-$, 459.3797 [M − H − Ac − 3Glc]$^-$
53	91.60	G-Ro	$C_{48}H_{76}O_{19}$	955.4908 ##	0.05	793.4388 [M − H − Glc]$^-$, 631.3843 [M − H − 2Glc]$^-$, 455.3520 [M − H − 2Glc − Glu A]$^-$
54	93.05	pseudo-G-RC$_1$ isomer	$C_{50}H_{84}O_{19}$	1033.5488 #	−0.92	987.5504 [M − H]$^-$, 945.5280 [M − H − Ac]$^-$, 783.4746 [M − H − Ac − Glc]$^-$, 621.4289 [M − H − Ac − 2Glc]$^-$, 459.3838 [M − H − Ac − 3Glc]$^-$
55	93.87	Ma-G-Rd/ isomer	$C_{51}H_{84}O_{21}$	1031.5375 ##	−0.50	987.5420 [M − H − CO$_2$]$^-$, 945.5335 [M − H − CO$_2$ − Ac]$^-$, 783.4844 [M − H − CO$_2$ − Ac − Glc]$^-$, 621.4318 [M − H − CO$_2$ − Ac − 2Glc]$^-$, 459.3841 [M − H − CO$_2$ − Ac − 3Glc]$^-$
56	94.20	chikusetsusaponin IV	$C_{47}H_{74}O_{18}$	925.4815 ##	0.19	763.4193 [M − H − Glc]$^-$, 631.3831 [M − H − Glc − Ara(f)]$^-$, 455.3536 [M − H − Glc − Xyl/Ara(f) − Glu A]$^-$
57	95.05	Ma-G-Rd/isomer	$C_{51}H_{84}O_{21}$	1031.5342 ##	−0.82	987.5497 [M − H − CO$_2$]$^-$, 945.5327 [M − H − CO$_2$ − Ac]$^-$, 783.4860 [M − H − CO$_2$ − Ac − Glc]$^-$, 621.4290 [M − H − CO$_2$ − Ac − 2Glc]$^-$, 459.3741 [M − H − CO$_2$ − Ac − 3Glc]$^-$
58	96.24	G-Rg$_3$	$C_{42}H_{72}O_{13}$	829.4934 #	−0.18	783.4813 [M − H]$^-$, 621.4281 [M − H − Glc]$^-$, 459.3775 [M − H − 2Glc]$^-$
59	99.43	chikusetsusaponin IVa	$C_{42}H_{66}O_{14}$	793.4344 ##	−0.38	631.3751 [M − H − Glc]$^-$, 455.3527 [M − H − Glc − Glu A]$^-$
60	100.93	pseudo-G-RT$_1$	$C_{47}H_{74}O_{18}$	925.4751 ##	−0.50	763.4211 [M − H − Glc]$^-$, 631.3892 [M − H − Glc − Xyl]$^-$, 455.3538 [M − H − Glc − Xyl − Glu A]$^-$

Stand for the measured value of [M + HCOO]$^-$ (*m*/*z*); ## Stand for the measured value of [M − H]$^-$ (*m*/*z*); The peaks **3–6**, **8–10**, **12–14**, **21**, **22**, **24–34**, **37**, **40**, **41**, **43**, **44**, **50**, **53**, **56**, **and 58** were characterized by comparing with the retention time (t_R)and fragmentation patterns of the their authentic standards.

Table S2. List of production areas of GRR.

No.	Origins	No.	Origins
1	Yangcha village, Taiwang town, Ji'an city [a]	25	Dongsheng nine feeder, Lushuihe, Fusong county [a]
2	Zhengyi village, Dalu town, Ji'an city [a]	26	Yongqing tree farm, Lushuihe, Fusong county [a]
3	Gumaling, Dalu town, Ji'an city [a]	27	Quanyanghezivillage, Quanyang town, Fusong county [a]
4	Yangzigou village, Yulin town, Ji'an city [a]	28	Dafang village, Wanliang town, Fusong county [a]
5	Liangshui township, Dongwan'gou, Ji'an city [a]	29	Xingshen town, Fusong county [a]
6	Haozigou village, Qingshi town, Ji'an city [a]	30	Guosong village, Donggang town, Fusong county [a]
7	Qiupi village, Qingshi town, Ji'an city [a]	31	Qinggouzi town, Dunhua city [a]
8	Mati village, Caiyuan town, Ji'an city [a]	32	Hanzhang town, Dunhua city [a]
9	Donggou village, Huadian town, Ji'an city [a]	33	Shangri village, Dunhua city [a]
10	Re'nao village, Qinghe town, Ji'an city [a]	34	Tianqiaoling Zhuanping, Wangqing county [a]
11	Qinggou village, Qinghe town, Ji'an city [a]	35	Xihe, Luozigou town, Wangqing county [a]
12	Xi village, Toudao town, Ji'an city [a]	36	Fuxing town, Wangqing county [a]
13	Louzigou village, Toudao town, Ji'an city [a]	37	Xiagou village thirteen group, Luozigou town, Wangqing county [a]
14	Shimiao village, Maxian township, Ji'an city [a]	38	Sidaohezi farm, Luozigou town, Wangqing county [a]
15	Shanghuolong village, Maxian township, Ji'an city [a]	39	Wangqing county [a]
16	Xintunzi town, Fusong county [a]	40	Fuxing village, Mengjiang town, Jingyu county [a]
17	Lushuihe Qingshuihe eastern mountain, Fusong county [a]	41	Six group, Yanping town, Jingyu county [a]
18	Beigang, Fusong county [a]	42	Mengjiang town, Jingyu county [a]
19	Xintunzi town, Fusong county [a]	43	Zhuanshanzi, Mengjiang town, Jingyu county [a]
20	Quanyanghezi village, Quanyang town, Fusong county [a]	44	Yanping town, Jingyu county [a]
21	Quanyang Jiangdong village, Fusong county [a]	45	Xujiadian village, Jingyu county [a]
22	Lushuihe Shalizihe village, Fusong county [a]	46	Sisea, Jingyu county [a]
23	Quanyang Quanshui, Fusong county [a]	47	Xinhe, Antu county [a]
24	Jianghe town, Fusong county [a]	48	Liangjiang town, Antu county [a]

Table S2. *Cont.*

No.	Origins	No.	Origins
49	Xinfangzi town, Changbai county [a]	58	Huashu village, Chunhua town, Huichun city [a]
50	Erdaogang village, Changbai county [a]	59	Dongxiaoshan farm, Linjiang city [a]
51	Liaohuangdi Baoquanshan, Changbai county [a]	60	Dongbeicha, Linjiang city [a]
52	Erdaogou, Changbai county [a]	61	Piaohe town, Jiaohe city [a]
53	Dading village, Xinfangzi town, Changbai county [a]	62	Changling farm, Qianjin town, Jiaohe city [a]
54	Majiagang village, Baoquanshan town, Changbai county [a]	63	Weidong forest farm 7 km forest zone [b]
55	Lenggouzi village, ShidIdaogou town, Changbai county [a]	64	Qitaihe city [b]
56	Dadingzi village, Xinfangzi town, Changbai county [a]	65	Gonghe town, Mudanjiang city [b]
57	Dading village, Xinfangzi town, Changbai county [a]	66	Qingan forestry bureau Jingou forest farm, Tieli city [b]

[a]: Cultivated in Jilin province of China; [b]: Cultivated in Heilongjiang province of China.

Table S3. The content of ginsenosides in 66 producing areas [a].

Analytes	The Content of Analytes (mg/g GRR)								
	1	2	3	4	5	6	7	8	9
G-Ra$_1$	0.437 ± 0.014	1.398 ± 0.079	0.408 ± 0.016	1.101 ± 0.007	0.907 ± 0.016	2.180 ± 0.006	2.424 ± 0.004	2.301 ± 0.001	1.353 ± 0.003
G-Ra$_2$	0.160 ± 0.011	0.831 ± 0.002	0.754 ± 0.008	0.618 ± 0.013	0.586 ± 0.016	1.202 ± 0.014	1.453 ± 0.003	1.233 ± 0.005	0.790 ± 0.006
G-Rb$_1$	2.050 ± 0.007	0.888 ± 0.017	4.078 ± 0.013	3.478 ± 0.001	7.678 ± 0.013	1.922 ± 0.008	1.901 ± 0.000	2.887 ± 0.011	3.558 ± 0.009
G-Rb$_2$	1.338 ± 0.015	0.647 ± 0.010	3.428 ± 0.018	2.310 ± 0.018	4.256 ± 0.012	0.933 ± 0.001	0.639 ± 0.015	1.269 ± 0.013	1.453 ± 0.011
G-Rb$_3$	0.520 ± 0.009	0.798 ± 0.020	0.580 ± 0.009	0.500 ± 0.011	1.379 ± 0.001	1.609 ± 0.008	1.232 ± 0.021	1.127 ± 0.019	1.053 ± 0.007
G-Rc	1.131 ± 0.002	0.885 ± 0.004	2.316 ± 0.010	2.021 ± 0.007	3.472 ± 0.006	1.500 ± 0.002	1.072 ± 0.001	1.766 ± 0.011	1.421 ± 0.007
G-Rd	0.995 ± 0.016	1.025 ± 0.005	1.777 ± 0.021	0.813 ± 0.018	1.418 ± 0.001	1.363 ± 0.018	0.875 ± 0.008	1.394 ± 0.029	0.893 ± 0.001
G-Re	1.758 ± 0.009	1.761 ± 0.006	2.679 ± 0.024	1.847 ± 0.004	4.661 ± 0.008	1.198 ± 0.012	2.980 ± 0.008	2.289 ± 0.003	2.252 ± 0.005
G-Rf	0.641 ± 0.009	0.405 ± 0.004	0.835 ± 0.001	0.312 ± 0.001	1.317 ± 0.005	0.645 ± 0.006	0.505 ± 0.006	0.761 ± 0.005	0.880 ± 0.003
G-Rg$_1$	2.160 ± 0.004	1.299 ± 0.017	3.410 ± 0.004	1.548 ± 0.003	4.477 ± 0.003	3.353 ± 0.003	1.516 ± 0.011	2.472 ± 0.023	3.018 ± 0.006
G-Ro	2.500 ± 0.001	0.811 ± 0.006	1.704 ± 0.026	1.231 ± 0.022	2.050 ± 0.002	1.781 ± 0.005	2.101 ± 0.013	1.526 ± 0.012	2.318 ± 0.003
20-glu-G-Rf	0.262 ± 0.007	0.115 ± 0.001	0.371 ± 0.005	0.194 ± 0.011	0.489 ± 0.011	0.374 ± 0.014	0.209 ± 0.000	0.277 ± 0.002	0.342 ± 0.006
NG-R$_1$	0.126 ± 0.005	0.154 ± 0.008	0.698 ± 0.012	0.667 ± 0.013	0.927 ± 0.017	0.684 ± 0.000	0.108 ± 0.002	0.068 ± 0.004	0.400 ± 0.000
NG-R$_2$	0.065 ± 0.002	0.164 ± 0.004	0.699 ± 0.010	0.485 ± 0.003	0.451 ± 0.001	0.402 ± 0.002	0.069 ± 0.002	0.048 ± 0.001	0.100 ± 0.005
Total content	14.143 ± 0.109	11.177 ± 0.184	23.736 ± 0.177	17.122 ± 0.135	34.068 ± 0.111	19.146 ± 0.100	17.084 ± 0.094	19.418 ± 0.139	19.832 ± 0.071

Table S3. *Cont.*

**Analytes**	**The Content of Analytes (mg/g GRR)**								
	**10**	**11**	**12**	**13**	**14**	**15**	**16**	**17**	**18**
G-Ra$_1$	1.030 ± 0.002	1.116 ± 0.002	1.906 ± 0.002	1.544 ± 0.012	1.807 ± 0.018	0.705 ± 0.013	0.560 ± 0.011	3.757 ± 0.006	2.018 ± 0.004
G-Ra$_2$	0.327 ± 0.005	0.810 ± 0.022	1.154 ± 0.014	0.751 ± 0.008	0.681 ± 0.005	0.261 ± 0.011	0.174 ± 0.006	1.786 ± 0.010	1.008 ± 0.020
G-Rb$_1$	2.800 ± 0.004	1.316 ± 0.008	2.533 ± 0.010	3.409 ± 0.001	2.900 ± 0.013	2.799 ± 0.010	1.802 ± 0.002	1.915 ± 0.013	4.168 ± 0.011
G-Rb$_2$	2.023 ± 0.016	0.324 ± 0.006	1.031 ± 0.010	2.660 ± 0.022	1.387 ± 0.008	1.302 ± 0.012	1.447 ± 0.013	1.414 ± 0.012	2.118 ± 0.006
G-Rb$_3$	0.473 ± 0.008	0.768 ± 0.009	1.315 ± 0.010	0.960 ± 0.017	0.610 ± 0.009	0.462 ± 0.011	0.449 ± 0.008	0.958 ± 0.009	0.525 ± 0.011
G-Rc	1.937 ± 0.003	0.539 ± 0.016	1.343 ± 0.008	2.847 ± 0.009	2.035 ± 0.009	1.366 ± 0.011	1.492 ± 0.016	2.257 ± 0.016	2.130 ± 0.000
G-Rd	1.008 ± 0.025	0.400 ± 0.001	0.911 ± 0.002	1.408 ± 0.014	1.295 ± 0.013	0.909 ± 0.012	0.647 ± 0.001	1.830 ± 0.001	1.751 ± 0.016
G-Re	1.875 ± 0.013	1.432 ± 0.020	2.410 ± 0.029	2.502 ± 0.020	1.971 ± 0.019	1.404 ± 0.011	1.329 ± 0.023	1.845 ± 0.005	1.933 ± 0.005
G-Rf	0.617 ± 0.011	0.431 ± 0.002	0.944 ± 0.006	0.885 ± 0.009	0.903 ± 0.008	0.742 ± 0.007	0.289 ± 0.003	0.534 ± 0.003	0.667 ± 0.004
G-Rg$_1$	1.880 ± 0.014	1.009 ± 0.010	2.690 ± 0.017	2.322 ± 0.009	1.700 ± 0.013	2.582 ± 0.014	1.054 ± 0.002	1.499 ± 0.018	3.132 ± 0.006
G-Ro	1.703 ± 0.018	1.170 ± 0.017	2.592 ± 0.009	2.848 ± 0.003	2.614 ± 0.011	1.766 ± 0.012	1.683 ± 0.005	1.239 ± 0.008	2.231 ± 0.012
20-glu-G-Rf	0.194 ± 0.009	0.158 ± 0.006	0.332 ± 0.014	0.317 ± 0.016	0.166 ± 0.004	0.250 ± 0.001	0.188 ± 0.004	0.214 ± 0.009	0.303 ± 0.002
NG-R$_1$	0.038 ± 0.001	0.146 ± 0.001	0.143 ± 0.003	0.344 ± 0.001	0.037 ± 0.001	0.044 ± 0.001	0.419 ± 0.004	0.332 ± 0.009	0.738 ± 0.004
NG-R$_2$	0.103 ± 0.004	0.039 ± 0.001	0.074 ± 0.000	0.441 ± 0.003	0.029 ± 0.002	0.025 ± 0.001	0.250 ± 0.002	0.259 ± 0.004	0.386 ± 0.000
Total content	16.005 ± 0.133	9.657 ± 0.121	19.379 ± 0.135	23.237 ± 0.143	18.133 ± 0.130	14.619 ± 0.135	11.783 ± 0.102	19.839 ± 0.123	23.108 ± 0.102
	**19**	**20**	**21**	**22**	**23**	**24**	**25**	**26**	
G-Ra$_1$	1.563 ± 0.007	1.537 ± 0.002	0.136 ± 0.007	3.168 ± 0.015	1.813 ± 0.012	0.976 ± 0.001	2.231 ± 0.011	0.112 ± 0.001	
G-Ra$_2$	0.693 ± 0.002	0.778 ± 0.005	0.071 ± 0.004	1.489 ± 0.010	0.718 ± 0.000	0.527 ± 0.013	1.087 ± 0.010	0.065 ± 0.001	
G-Rb$_1$	4.254 ± 0.003	3.815 ± 0.001	2.821 ± 0.017	2.559 ± 0.006	5.826 ± 0.021	2.653 ± 0.004	4.299 ± 0.006	5.087 ± 0.012	
G-Rb$_2$	2.914 ± 0.012	1.494 ± 0.015	2.802 ± 0.012	1.707 ± 0.014	4.673 ± 0.005	1.912 ± 0.013	2.328 ± 0.002	3.774 ± 0.011	
G-Rb$_3$	0.667 ± 0.002	0.696 ± 0.008	0.513 ± 0.012	1.250 ± 0.009	1.074 ± 0.010	0.582 ± 0.012	0.734 ± 0.019	0.516 ± 0.012	
G-Rc	2.693 ± 0.016	1.836 ± 0.009	2.211 ± 0.014	1.750 ± 0.012	4.635 ± 0.007	1.658 ± 0.009	2.685 ± 0.007	3.401 ± 0.010	
G-Rd	2.211 ± 0.019	1.247 ± 0.018	1.599 ± 0.016	3.397 ± 0.023	2.860 ± 0.005	1.327 ± 0.012	1.684 ± 0.013	1.356 ± 0.016	
G-Re	1.802 ± 0.010	1.754 ± 0.015	1.462 ± 0.010	2.469 ± 0.003	2.821 ± 0.017	1.858 ± 0.003	2.079 ± 0.002	3.028 ± 0.013	
G-Rf	0.677 ± 0.009	0.472 ± 0.007	0.809 ± 0.008	1.435 ± 0.001	1.127 ± 0.011	0.828 ± 0.001	1.046 ± 0.003	0.833 ± 0.010	
G-Rg$_1$	1.986 ± 0.674	2.216 ± 0.019	2.940 ± 0.011	2.784 ± 0.017	3.050 ± 0.004	2.287 ± 0.019	3.222 ± 0.008	3.202 ± 0.017	
G-Ro	2.174 ± 0.009	2.067 ± 0.014	3.818 ± 0.008	1.692 ± 0.011	5.693 ± 0.005	2.630 ± 0.004	4.551 ± 0.004	2.277 ± 0.013	
20-glu-G-Rf	0.322 ± 0.012	0.272 ± 0.002	0.364 ± 0.009	0.309 ± 0.011	0.433 ± 0.007	0.266 ± 0.004	0.335 ± 0.012	0.396 ± 0.004	
NG-R$_1$	0.695 ± 0.005	0.578 ± 0.001	0.326 ± 0.010	0.040 ± 0.005	0.098 ± 0.002	0.060 ± 0.004	0.343 ± 0.000	0.725 ± 0.009	
NG-R$_2$	0.553 ± 0.007	0.388 ± 0.004	0.287 ± 0.005	0.045 ± 0.001	0.059 ± 0.003	0.097 ± 0.001	0.269 ± 0.009	0.570 ± 0.014	
Total content	23.205 ± 0.788	19.150 ± 0.121	20.158 ± 0.144	24.094 ± 0.140	34.880 ± 0.109	17.661 ± 0.099	26.894 ± 0.107	25.342 ± 0.143	

Table S3. *Cont.*

Analytes	The Content of Analytes (mg/g GRR)							
	**27**	**28**	**29**	**30**	**31**	**32**	**33**	**34**
G-Ra1	1.162 ± 0.013	0.412 ± 0.002	0.942 ± 0.023	1.851 ± 0.022	1.084 ± 0.004	0.821 ± 0.012	1.652 ± 0.011	1.530 ± 0.012
G-Ra2	0.416 ± 0.008	0.178 ± 0.001	0.400 ± 0.004	0.737 ± 0.019	0.507 ± 0.005	0.279 ± 0.006	0.741 ± 0.012	0.824 ± 0.012
G-Rb1	7.723 ± 0.012	5.224 ± 0.006	1.414 ± 0.006	7.572 ± 0.019	3.916 ± 0.018	3.236 ± 0.005	1.807 ± 0.017	2.143 ± 0.010
G-Rb2	5.261 ± 0.005	3.532 ± 0.006	0.576 ± 0.001	5.555 ± 0.010	3.251 ± 0.007	2.800 ± 0.017	0.925 ± 0.010	1.155 ± 0.011
G-Rb3	0.871 ± 0.012	0.619 ± 0.014	0.303 ± 0.008	1.154 ± 0.011	0.483 ± 0.007	0.516 ± 0.006	0.547 ± 0.011	0.763 ± 0.012
G-Rc	5.089 ± 0.000	2.508 ± 0.014	0.529 ± 0.005	5.029 ± 0.011	2.851 ± 0.009	2.607 ± 0.015	1.210 ± 0.018	1.149 ± 0.010
G-Rd	3.690 ± 0.013	0.992 ± 0.028	0.686 ± 0.015	3.674 ± 0.024	1.502 ± 0.015	2.324 ± 0.012	1.215 ± 0.014	0.711 ± 0.012
G-Re	2.824 ± 0.004	2.234 ± 0.025	1.063 ± 0.005	3.314 ± 0.001	2.557 ± 0.003	0.539 ± 0.002	2.393 ± 0.009	1.969 ± 0.021
G-Rf	1.060 ± 0.011	0.428 ± 0.004	0.349 ± 0.002	1.577 ± 0.010	0.593 ± 0.007	0.812 ± 0.004	0.469 ± 0.011	0.717 ± 0.009
G-Rg1	3.952 ± 0.001	2.667 ± 0.003	1.734 ± 0.005	4.189 ± 0.017	1.619 ± 0.003	2.472 ± 0.017	1.509 ± 0.014	2.408 ± 0.012
G-Ro	2.844 ± 0.008	2.445 ± 0.017	0.667 ± 0.002	3.718 ± 0.018	1.252 ± 0.010	1.930 ± 0.018	0.908 ± 0.012	1.348 ± 0.013
20-glu-G-Rf	0.497 ± 0.004	0.200 ± 0.008	0.115 ± 0.004	0.507 ± 0.021	0.190 ± 0.009	0.252 ± 0.003	0.196 ± 0.003	0.296 ± 0.004
NG-R1	0.523 ± 0.024	0.696 ± 0.019	0.238 ± 0.001	0.064 ± 0.006	0.559 ± 0.004	0.036 ± 0.003	0.177 ± 0.005	0.185 ± 0.007
NG-R2	0.642 ± 0.001	0.482 ± 0.001	0.320 ± 0.001	0.025 ± 0.001	0.196 ± 0.001	0.042 ± 0.002	0.140 ± 0.006	0.120 ± 0.006
Total content	36.552 ± 0.117	22.618 ± 0.146	9.338 ± 0.082	38.965 ± 0.190	20.561 ± 0.102	18.667 ± 0.124	13.889 ± 0.152	15.318 ± 0.153
	**35**	**36**	**37**	**38**	**39**	**40**	**41**	**42**
G-Ra1	1.007 ± 0.026	3.108 ± 0.006	1.174 ± 0.013	5.326 ± 0.007	2.879 ± 0.010	0.454 ± 0.013	2.536 ± 0.008	1.388 ± 0.010
G-Ra2	0.441 ± 0.015	1.672 ± 0.020	0.510 ± 0.008	2.656 ± 0.009	1.333 ± 0.020	0.151 ± 0.007	1.619 ± 0.011	0.693 ± 0.006
G-Rb1	3.929 ± 0.016	1.280 ± 0.003	6.675 ± 0.012	3.095 ± 0.010	2.477 ± 0.006	2.147 ± 0.014	2.166 ± 0.020	3.024 ± 0.009
G-Rb2	2.265 ± 0.066	0.592 ± 0.001	4.213 ± 0.012	1.387 ± 0.020	1.094 ± 0.000	2.234 ± 0.013	0.598 ± 0.018	1.319 ± 0.008
G-Rb3	0.579 ± 0.020	0.645 ± 0.013	0.615 ± 0.009	0.628 ± 0.018	0.558 ± 0.009	0.380 ± 0.013	0.362 ± 0.004	0.657 ± 0.026
G-Rc	2.285 ± 0.013	1.133 ± 0.005	3.176 ± 0.015	1.284 ± 0.008	1.485 ± 0.009	2.054 ± 0.012	0.621 ± 0.007	1.174 ± 0.020
G-Rd	1.745 ± 0.020	1.442 ± 0.007	3.657 ± 0.023	3.579 ± 0.022	1.686 ± 0.002	1.600 ± 0.010	0.922 ± 0.002	1.257 ± 0.021
G-Re	2.417 ± 0.014	2.288 ± 0.005	3.025 ± 0.005	3.327 ± 0.016	2.382 ± 0.014	1.636 ± 0.016	2.080 ± 0.003	1.187 ± 0.016
G-Rf	0.847 ± 0.014	0.709 ± 0.007	1.021 ± 0.004	0.968 ± 0.007	0.690 ± 0.008	0.745 ± 0.013	0.598 ± 0.009	0.890 ± 0.008
G-Rg1	2.950 ± 0.013	1.379 ± 0.009	3.207 ± 0.015	2.039 ± 0.007	2.264 ± 0.029	2.326 ± 0.012	1.644 ± 0.001	2.712 ± 0.024
G-Ro	2.010 ± 0.017	2.252 ± 0.016	2.395 ± 0.018	2.069 ± 0.016	1.075 ± 0.001	2.203 ± 0.017	4.611 ± 0.017	1.396 ± 0.006
20-glu-G-Rf	0.335 ± 0.010	0.108 ± 0.004	0.370 ± 0.014	0.248 ± 0.001	0.197 ± 0.004	0.382 ± 0.011	0.235 ± 0.010	0.331 ± 0.007
NG-R1	0.407 ± 0.008	0.048 ± 0.003	0.515 ± 0.013	0.028 ± 0.004	0.105 ± 0.006	0.206 ± 0.009	0.060 ± 0.003	0.091 ± 0.007
NG-R2	0.560 ± 0.003	0.033 ± 0.000	0.471 ± 0.005	0.086 ± 0.001	0.060 ± 0.000	0.289 ± 0.004	0.083 ± 0.000	0.037 ± 0.002
Total content	21.773 ± 0.255	16.688 ± 0.100	31.024 ± 0.166	26.722 ± 0.145	18.284 ± 0.119	16.803 ± 0.165	18.136 ± 0.114	16.157 ± 0.170

Table S3. *Cont.*

Analytes	The Content of Analytes (mg/g GRR)							
	**43**	**44**	**45**	**46**	**47**	**48**	**49**	**50**
G-Ra1	0.736 ± 0.013	0.531 ± 0.018	1.013 ± 0.018	1.230 ± 0.022	1.153 ± 0.014	0.839 ± 0.010	0.127 ± 0.005	2.084 ± 0.019
G-Ra2	0.239 ± 0.007	0.266 ± 0.005	0.534 ± 0.009	0.691 ± 0.023	0.698 ± 0.017	0.391 ± 0.008	0.110 ± 0.006	1.224 ± 0.019
G-Rb1	2.320 ± 0.016	3.942 ± 0.006	2.507 ± 0.016	3.738 ± 0.006	1.142 ± 0.017	3.914 ± 0.012	3.822 ± 0.026	2.553 ± 0.014
G-Rb2	2.835 ± 0.015	3.399 ± 0.009	1.881 ± 0.007	2.757 ± 0.012	0.275 ± 0.004	3.430 ± 0.015	3.838 ± 0.008	1.661 ± 0.003
G-Rb3	0.589 ± 0.008	0.586 ± 0.002	0.605 ± 0.009	0.686 ± 0.012	0.336 ± 0.009	0.846 ± 0.010	0.421 ± 0.013	0.706 ± 0.013
G-Rc	2.165 ± 0.009	3.118 ± 0.001	1.868 ± 0.017	2.599 ± 0.019	0.560 ± 0.019	3.214 ± 0.016	2.903 ± 0.019	1.304 ± 0.017
G-Rd	1.910 ± 0.017	2.382 ± 0.006	1.127 ± 0.019	1.325 ± 0.003	0.456 ± 0.009	1.909 ± 0.017	1.833 ± 0.020	1.126 ± 0.020
G-Re	1.661 ± 0.014	2.310 ± 0.009	2.207 ± 0.018	2.716 ± 0.004	1.228 ± 0.015	2.148 ± 0.015	1.409 ± 0.015	2.087 ± 0.033
G-Rf	0.496 ± 0.002	0.584 ± 0.005	0.610 ± 0.009	1.276 ± 0.007	0.551 ± 0.011	1.016 ± 0.011	0.741 ± 0.011	0.571 ± 0.001
G-Rg1	1.634 ± 0.006	2.342 ± 0.008	1.565 ± 0.013	3.888 ± 0.013	1.923 ± 0.013	2.974 ± 0.010	3.016 ± 0.015	1.672 ± 0.000
G-Ro	1.050 ± 0.015	2.204 ± 0.024	1.623 ± 0.019	3.881 ± 0.017	1.570 ± 0.010	2.422 ± 0.016	2.400 ± 0.009	5.030 ± 0.024
20-glu-G-Rf	0.137 ± 0.001	0.293 ± 0.003	0.239 ± 0.009	0.368 ± 0.008	0.271 ± 0.004	0.392 ± 0.011	0.432 ± 0.013	0.261 ± 0.005
NG-R1	0.135 ± 0.002	0.509 ± 0.004	0.295 ± 0.009	0.072 ± 0.000	0.518 ± 0.002	0.033 ± 0.002	0.827 ± 0.014	0.440 ± 0.008
NG-R2	0.129 ± 0.003	0.440 ± 0.008	0.211 ± 0.006	0.154 ± 0.001	0.139 ± 0.002	0.048 ± 0.002	0.648 ± 0.001	0.233 ± 0.010
Total content	16.035 ± 0.129	22.095 ± 0.107	16.286 ± 0.178	25.380 ± 0.147	10.818 ± 0.146	23.574 ± 0.156	22.526 ± 0.178	20.952 ± 0.186
	**51**	**52**	**53**	**54**	**55**	**56**	**57**	**58**
G-Ra1	3.159 ± 0.021	2.518 ± 0.017	2.065 ± 0.008	1.768 ± 0.016	1.397 ± 0.003	1.820 ± 0.016	0.598 ± 0.011	1.597 ± 0.005
G-Ra2	1.382 ± 0.008	1.203 ± 0.011	0.933 ± 0.005	1.115 ± 0.011	0.762 ± 0.010	1.002 ± 0.014	0.249 ± 0.005	0.779 ± 0.001
G-Rb1	5.992 ± 0.019	3.222 ± 0.009	5.536 ± 0.013	3.675 ± 0.013	3.711 ± 0.004	2.930 ± 0.003	4.490 ± 0.004	3.636 ± 0.013
G-Rb2	3.110 ± 0.020	2.098 ± 0.014	2.764 ± 0.014	0.817 ± 0.014	2.991 ± 0.013	1.543 ± 0.013	3.101 ± 0.009	2.287 ± 0.009
G-Rb3	1.129 ± 0.015	1.215 ± 0.016	0.742 ± 0.001	0.828 ± 0.012	0.490 ± 0.004	0.470 ± 0.004	0.625 ± 0.009	0.616 ± 0.007
G-Rc	3.207 ± 0.010	2.101 ± 0.011	2.807 ± 0.008	1.393 ± 0.010	2.882 ± 0.016	1.447 ± 0.012	2.907 ± 0.005	2.481 ± 0.005
G-Rd	2.816 ± 0.016	2.463 ± 0.011	2.108 ± 0.002	0.974 ± 0.018	2.641 ± 0.008	1.288 ± 0.011	1.670 ± 0.019	2.135 ± 0.007
G-Re	3.217 ± 0.020	3.459 ± 0.015	2.184 ± 0.009	1.385 ± 0.020	2.367 ± 0.005	1.599 ± 0.018	2.052 ± 0.001	1.961 ± 0.002
G-Rf	0.600 ± 0.010	0.994 ± 0.009	0.847 ± 0.009	1.277 ± 0.010	0.841 ± 0.004	0.526 ± 0.007	0.837 ± 0.000	0.969 ± 0.005
G-Rg1	2.148 ± 0.012	2.399 ± 0.010	2.763 ± 0.005	3.876 ± 0.014	2.640 ± 0.006	1.974 ± 0.013	2.616 ± 0.007	3.662 ± 0.009
G-Ro	4.924 ± 0.023	3.572 ± 0.014	3.421 ± 0.006	2.927 ± 0.018	2.895 ± 0.013	2.704 ± 0.010	1.856 ± 0.017	2.284 ± 0.012
20-glu-G-Rf	0.401 ± 0.000	0.354 ± 0.004	0.312 ± 0.001	0.520 ± 0.008	0.316 ± 0.002	0.270 ± 0.001	0.195 ± 0.016	0.265 ± 0.010
NG-R1	0.943 ± 0.032	0.536 ± 0.007	0.654 ± 0.017	0.334 ± 0.012	0.818 ± 0.007	0.649 ± 0.002	0.266 ± 0.003	0.026 ± 0.002
NG-R2	0.401 ± 0.003	0.476 ± 0.004	0.240 ± 0.005	0.069 ± 0.004	0.579 ± 0.012	0.295 ± 0.007	0.426 ± 0.010	0.071 ± 0.001
Total content	33.430 ± 0.210	26.610 ± 0.151	27.376 ± 0.102	20.959 ± 0.181	25.329 ± 0.110	18.518 ± 0.131	21.886 ± 0.118	22.770 ± 0.087

Table S3. *Cont.*

Analytes	The Content of Analytes (mg/g GRR)							
	**59**	**60**	**61**	**62**	**63**	**64**	**65**	**66**
G-Ra$_1$	2.625 ± 0.010	0.116 ± 0.005	1.501 ± 0.018	0.717 ± 0.011	1.546 ± 0.001	1.555 ± 0.016	0.555 ± 0.014	1.010 ± 0.007
G-Ra$_2$	1.563 ± 0.016	0.165 ± 0.003	0.941 ± 0.004	0.319 ± 0.015	0.482 ± 0.003	0.917 ± 0.015	0.215 ± 0.007	0.383 ± 0.015
G-Rb$_1$	2.916 ± 0.013	6.721 ± 0.335	1.187 ± 0.011	3.867 ± 0.012	3.754 ± 0.009	2.455 ± 0.019	1.715 ± 0.014	4.648 ± 0.017
G-Rb$_2$	1.119 ± 0.010	8.247 ± 0.018	0.494 ± 0.009	2.047 ± 0.013	3.289 ± 0.003	1.417 ± 0.014	1.790 ± 0.013	4.275 ± 0.006
G-Rb$_3$	1.041 ± 0.007	1.155 ± 0.003	0.760 ± 0.009	0.485 ± 0.017	0.743 ± 0.011	0.734 ± 0.015	0.559 ± 0.006	0.773 ± 0.005
G-Rc	1.732 ± 0.015	6.122 ± 0.019	0.774 ± 0.013	2.146 ± 0.013	3.226 ± 0.005	1.856 ± 0.018	1.740 ± 0.017	4.601 ± 0.001
G-Rd	1.089 ± 0.015	4.338 ± 0.013	0.485 ± 0.006	1.027 ± 0.020	3.507 ± 0.011	1.199 ± 0.010	0.817 ± 0.013	1.983 ± 0.007
G-Re	1.993 ± 0.012	4.815 ± 0.001	1.396 ± 0.017	1.669 ± 0.011	2.870 ± 0.008	1.579 ± 0.021	1.578 ± 0.008	2.512 ± 0.011
G-Rf	1.123 ± 0.009	1.369 ± 0.005	0.352 ± 0.009	0.790 ± 0.006	0.833 ± 0.007	0.767 ± 0.007	0.297 ± 0.004	0.471 ± 0.010
G-Rg$_1$	3.072 ± 0.013	6.095 ± 0.018	1.028 ± 0.019	2.675 ± 0.015	2.463 ± 0.001	2.545 ± 0.016	0.870 ± 0.010	1.656 ± 0.017
G-Ro	5.354 ± 0.014	6.959 ± 0.018	1.820 ± 0.011	1.717 ± 0.015	1.156 ± 0.013	1.530 ± 0.013	1.958 ± 0.019	1.692 ± 0.022
20-glu-G-Rf	0.358 ± 0.002	0.739 ± 0.016	0.181 ± 0.002	0.304 ± 0.008	0.215 ± 0.005	0.338 ± 0.012	0.133 ± 0.004	0.185 ± 0.002
NG-R$_1$	0.084 ± 0.005	1.185 ± 0.007	0.161 ± 0.004	0.067 ± 0.004	0.298 ± 0.002	0.168 ± 0.005	0.105 ± 0.001	0.331 ± 0.006
NG-R$_2$	0.042 ± 0.003	0.599 ± 0.002	0.112 ± 0.005	0.024 ± 0.002	0.552 ± 0.007	0.115 ± 0.004	0.084 ± 0.002	0.243 ± 0.010
Total content	24.110 ± 0.144	48.624 ± 0.464	11.191 ± 0.136	17.854 ± 0.162	24.931 ± 0.087	17.175 ± 0.185	12.415 ± 0.134	24.762 ± 0.137

[a]. Analysis was performed in triplicate.

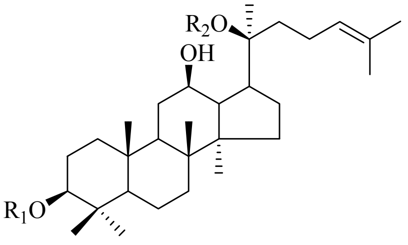

	R$_1$	R$_2$
G-Ra$_1$	-Glc^{2}-^{1}Glc	-Glc^{6}-^{1}Ara(p)^{4}-^{1}Xyl
G-Ra$_2$	-Glc^{2}-^{1}Glc	-Glc^{6}-^{1}Ara(f)^{2}-^{1}Xyl
G-Ra$_3$	-Glc^{2}-^{1}Glc	-Glc^{6}-^{1}Glc^{3}-^{1}Xyl
G-Rb$_1$	-Glc^{2}-^{1}Glc	-Glc^{6}-^{1}Glc
G-Rb$_2$	-Glc^{2}-^{1}Glc	-Glc^{6}-^{1}Ara(p)
Ma-G-Rb$_2$	-Glc^{2}-^{1}Glc^{6}-malonyl	-Glc^{6}-^{1}Ara(p)
G-Rc	-Glc^{2}-^{1}Glc	-Glc^{6}-^{1}Ara(f)
G-Rb$_3$	-Glc^{2}-^{1}Glc	-Glc^{6}-^{1}Xyl
G-Rd	-Glc^{2}-^{1}Glc	-Glc
G-Rg$_3$	-Glc^{2}-^{1}Glc	-H
G-Rs$_1$	-Glc^{2}-^{1}Glc^{6}Ac	-Glc^{6}-^{1}Ara(p)
G-Rs$_2$	-Glc^{2}-^{1}Glc^{6}Ac	-Glc^{6}-^{1}Ara(f)
NG-R$_4$	-Glc^{2}-^{1}Glc	-Glc^{6}-^{1}Glc^{6}-^{1}Xyl
PQ-R$_1$	-Glc^{2}-^{1}Glc^{6}Ac	-Glc^{6}-^{1}Glc

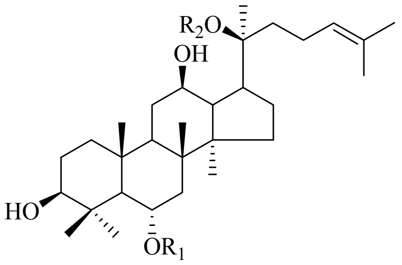

	R$_1$	R$_2$
G-Re	-Glc^{2}-^{1}Rha	-Glc
G-Re$_1$	-Glc	-Glc^{3}-^{1}Glc
G-Re$_2$	-Glc^{3}-^{1}Glc	-Glc
G-Re$_3$	-Glc	-Glc^{4}-^{1}Glc
G-Re$_4$	-Glc	-Glc^{6}-^{1}Ara(f)
G-Rf	-Glc^{2}-^{1}Glc	-H
20-glc-G-Rf	-Glc^{2}-^{1}Glc	-Glc
G-Rg$_1$	-Glc	-Glc
G-Rg$_2$	-Glc^{2}-^{1}Rha	-H
G-Rh$_1$	-Glc	-H
KG-R$_1$	-Glc^{6}-(*E*)-but-2-enoyl	-Glc
NG-N	-Glc^{4}-^{1}Glc	-Glc
NG-R$_1$	-Glc^{2}-^{1}Xyl	-Glc
NG-R$_2$	-Glc^{2}-^{1}Xyl	-H

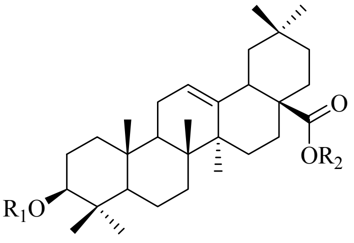

	R$_1$	R$_2$
oleanolic acid	-H	-H
G-Ro	Glc^{1}-^{2}Glu A-	-Glc
G-RoMe	Glc^{1}-^{2}Glu A^{6}-Me ester-	-Glc
chikusetsusaponin IV	Ara(f)^{1}-^{4}Glu A-	-H

Figure S1. Chemical structures of ginsenosides.
